# A New Nanobody-Based Biosensor to Study Endogenous PARP1 *In Vitro* and in Live Human Cells

**DOI:** 10.1371/journal.pone.0151041

**Published:** 2016-03-07

**Authors:** Andrea Buchfellner, Larisa Yurlova, Stefan Nüske, Armin M. Scholz, Jacqueline Bogner, Benjamin Ruf, Kourosh Zolghadr, Sophie E. Drexler, Guido A. Drexler, Stefanie Girst, Christoph Greubel, Judith Reindl, Christian Siebenwirth, Tina Romer, Anna A. Friedl, Ulrich Rothbauer

**Affiliations:** 1 ChromoTek GmbH, IZB, Planegg, Martinsried, Germany; 2 Livestock Center of the Faculty of Veterinary Medicine, Ludwig Maximilians University, Munich, Germany; 3 Department of Radiation Oncology, Ludwig Maximilians University, Munich, Germany; 4 Institute for Applied Physics and Metrology, Bundeswehr University Munich, Neubiberg, Germany; 5 Department of Radiation Oncology, Klinikum rechts der Isar, Technical University Munich, Munich, Germany; 6 Clinical Cooperation Group ‘Personalized Radiotherapy of Head and Neck Cancer’, Helmholtz Center Munich, Neuherberg, Germany; 7 Natural and Medical Institute at the University of Tuebingen, Reutlingen, Germany; 8 Pharmaceutical Biotechnology, University of Tuebingen, Tuebingen, Germany; University of Pecs Medical School, HUNGARY

## Abstract

Poly(ADP-ribose) polymerase 1 (PARP1) is a key player in DNA repair, genomic stability and cell survival and it emerges as a highly relevant target for cancer therapies. To deepen our understanding of PARP biology and mechanisms of action of PARP1-targeting anti-cancer compounds, we generated a novel PARP1-affinity reagent, active both *in vitro* and in live cells. This PARP1-biosensor is based on a PARP1-specific single-domain antibody fragment (~ 15 kDa), termed nanobody, which recognizes the N-terminus of human PARP1 with nanomolar affinity. In proteomic approaches, immobilized PARP1 nanobody facilitates quantitative immunoprecipitation of functional, endogenous PARP1 from cellular lysates. For cellular studies, we engineered an intracellularly functional PARP1 chromobody by combining the nanobody coding sequence with a fluorescent protein sequence. By following the chromobody signal, we were for the first time able to monitor the recruitment of endogenous PARP1 to DNA damage sites in live cells. Moreover, tracing of the sub-nuclear translocation of the chromobody signal upon treatment of human cells with chemical substances enables real-time profiling of active compounds in high content imaging. Due to its ability to perform as a biosensor at the endogenous level of the PARP1 enzyme, the novel PARP1 nanobody is a unique and versatile tool for basic and applied studies of PARP1 biology and DNA repair.

## Introduction

Poly(ADP-ribose) polymerase (PARP) proteins are involved in DNA repair, gene expression regulation, genomic stability and cell death. Human PARP family comprises 17 members, out of which PARP1 is the most abundant and best characterized. Due to its critical role in the repair processes of DNA strand breaks, PARP1 became an important target for drug discovery in cancer therapeutics. Human PARP1 is a 113 kDa protein consisting of three main domains: an N-terminal DNA-binding domain (containing three zinc fingers) [1, 2], a central automodification domain and a C-terminal catalytic domain [3, 4].

Upon DNA damage, PARP1 is recruited to DNA lesions [5], where it binds DNA through its N-terminal zinc finger motives [6]. Subsequently, PARP1 mediates the process of PARylation using nicotinamide adenine dinucleotide (NAD+) as a substrate to catalyze the covalent transfer of ADP-ribose units to a variety of nuclear acceptor proteins such as transcription factors, histones, DNA repair enzymes and PARP1 itself [7, 8]. This PARylation triggers local relaxation of the chromatin structure and recruitment of the DNA repair machinery (XRCC1, DNA ligase III, DNA polymerase ß, Ku70) [9].

Blocking DNA repair is an attractive strategy for sensitizing cancer cells to radio- and/or chemotherapy, and being at the initiating point of the DNA repair cascades, PARP1 is a valid target for these strategies. Several PARP-specific inhibitors have been developed up to date; including niraparib (MK-4827), olaparib (AZD-2281) and veliparib (ABT-888) which are currently tested in clinical studies. These inhibitors are especially potent when applied to breast cancer gene (BRCA) deficient cells, in which they induce synthetic cytotoxicity [10]. However, the results of the clinical studies are so far contradictory. Furthermore, the molecular mechanisms of action of the PARP-targeting compounds (e.g. catalytic inhibition, or additional PARP1-“trapping”) require additional investigation.

Due to the utmost importance of understanding the biology of PARP for unraveling the principles of DNA repair and for developing cancer-targeting therapies, there is ongoing need for reliable research tools addressing PARP1 dynamics. So far, common approaches for microscopy-based examination of PARP localization and dynamics rely on staining of endogenous PARP1 with specific antibodies in fixed cells or on heterologous expression of chimeric fluorescent fusion constructs (e.g. GFP-PARP1). Notably, immunostaining procedures are not free from aberrations or artifacts, depending on the fixation and permeabilization methods and on the antibodies of choice [11, 12]. This problem is especially relevant for PARP detection, as several PARP-specific antibodies have shown different subnuclear localization at different concentrations of PFA [13–16]. On the other hand, ectopically expressed fluorescent PARP1-fusion proteins might not reflect the behavior of their endogenous counterpart. Overexpression of PARP1 changes the intracellular PARP1 level and therefore might have an impact on PARP1 cellular distribution and function.

Taken together, until now there was no tool available which would enable live-cell detection of endogenous PARP1. To overcome this technical limitation, we took advantage of single-domain camelid antibodies. Heavy-chain only antibodies contain the smallest naturally occurring antigen-binding domain, which is comprised of only one polypeptide chain. This domain is termed variable domain of heavy-chain antibodies (V_H_H), or simply “nanobody”. The advantage of nanobodies lies in their single-domain nature, stability, solubility and small size. These binding molecules are only 15 kDa in size and functional in the reducing environment of the cytoplasm, as has been recently shown [17–20].

Here, we focused on the characterization of a newly developed PARP1-specific nanobody and on its performance in the following techniques and applications: immunoprecipitation, live-cell imaging and high content analysis (HCA). We discuss the advantages of the PARP1 nanobody compared to conventional PARP1 immunoreagents in the tested applications. Furthermore, we demonstrate that our PARP1 nanobody enables live-cell immunodetection of endogenous PARP1 dynamics, previously not possible with existing reagents and methods.

## Materials and Methods

### V_H_H library and screening

One alpaca (*Vicugna pacos*) was immunized with purified autoPARylated hPARP1 protein according to the protocol described previously [21]. Alpacas belong to Livestock Center of the Faculty of Veterinary Medicine, Ludwig Maximilians University, Munich. Immunization was performed in strict accordance with the German Animal Welfare Law and has been approved by the government of Upper Bavaria (Permit number: 55.2-1-54-2531.6-9-06). 70 days after the first immunization, ~100 ml blood was collected from the animal and lymphocytes were isolated by Ficoll gradient centrifugation using the Lymphocyte Separation Medium (PAA Laboratories GmbH). 1x 10^7^ B-cells were used to prepare total RNA using the Nucleospin RNA Kit (Macherey-Nagel). Complementary DNA (cDNA) was amplified using the First-Strand cDNA Synthesis Kit (GE Healthcare) according to the manufacturer´s protocol. The V_H_H repertoire was amplified from the cDNA by 3 subsequent nested PCR reactions using 6 different V_H_H-specific primers [17]. The V_H_H library was subcloned into the *Sfi*I/*Not*I sites of the pHEN4 phagemid vector and transformed into *E*. *coli* TG1 cells [22]. *E*. *coli* cells were further infected with M13K07 helper phages to produce phages carrying V_H_Hs on their tips. The phage display/immunopanning procedures and ELISA were performed as detailed in [23]. For further studies, a V_H_H with the highest solubility and affinity to PARP1 was selected.

### Expression plasmids

For bacterial expression of the V_H_H domain (nanobody), the sequence were cloned into the pHEN6 vector [22], thereby adding a C-terminal 6xHis-tag for IMAC purification. Bacterial expression vector of PARP1 V_H_H will be provided upon request to the authors by ChromoTek via MTA (material transfer agreement). For protein production, *E*. *coli* JM109 cells (NEB) were used. Expression and purification of the nanobody was carried out as described previously [24]. For mammalian expression of PARP1 chromobody, N-terminal fusions of the PARP1 nanobody to the fluorescent proteins TagGFP2 or TagRFP (Evrogen) were constructed using *Bgl*II/*Hin*dIII restriction sites in the target backbone vector. PARP1 Chromobody vector will be provided upon request to the authors by ChromoTek via MTA. All resulting constructs were sequenced and tested for expression in HEK293T cells followed by immunoblot analysis. Mammalian expression plasmids of GFP-hPARP1, GFP-hPARP2, GFP-hPARP3 and GFP-hPARP9 were kindly provided by Prof. Heinrich Leonhardt, LMU Munich. The plasmids coding for the GFP- or mCherry-tagged PARP1 domains (DBD-GFP, ZnF1-GFP, ZnF2-GFP, mCherry-ZnF3, WGR-PARP domain-mCherry) were kindly provided by Gyula Timinszky, LMU Munich. SF9 insect cells expressing Strep-tagged human PARP1 domains (DNA-binding domain, automodification domain, catalytic domain) where kindly provided by Annette Becker, TU Darmstadt. Point mutations were introduced into the human ZnF2 sequence with the Q5^®^ site-directed mutagenesis kit (NEB) according to the manufacturer’s instructions.

### Antibodies and chemical compounds

The following primary antibodies were used: rat anti-GFP clone 3H9 (ChromoTek), mouse anti-RFP clone 3F5 (ChromoTek), rabbit anti-TagRFP (Evrogen, AB233), mouse anti-PARP1 clone CII-10 (BD-Biosciences), mouse anti-pADPr clone 10H (Santa Cruz) and rabbit anti-GAPDH antibody (Santa Cruz, sc 25778). The following secondary antibodies were used for detecting the primaries: anti-rat/mouse/rabbit-Alexa Fluor 647/568/488 (Cell Signaling). The following small molecule compounds were administered: camptothecin (Tocris), actinomycin D (Sigma), 4-NQO (Sigma) and H_2_O_2_ (Sigma). The following affinity resins were used for immunoprecipitation: GFP-Trap^®^, RFP-Trap^®^ and PARP1 nanotrap (ChromoTek).

### PARP1-affinity resin generation and immunoprecipitation

Purified V_H_H was covalently coupled to Sepharose beads (GE Healthcare) via NHS according to the manufacturer´s protocol, creating so-called PARP1 nanotrap. For immunoprecipitation, 1 x 10^6^–1 x 10^7^ HEK293T, HeLa, MEF or BHK cells expressing the target protein were washed and harvested in phosphate buffered saline (PBS). Cell pellets were homogenized in 200 μl RIPA buffer (10 mM Tris/Cl pH7.5, 150 mM NaCl, 0.5 mM EDTA, 0.1% SDS, 1% Triton X-100, 1% Deoxycholate), supplemented with 1 μg/μl DNaseI, 2 mM MgCl_2_, 2 mM PMSF, 1x mammalian protease inhibitor mix M (Serva) by repeated pipetting for 30 min on ice. After a centrifugation step (10 min at 17.000 x g), the soluble fraction was adjusted to 500 μl with a dilution buffer (10 mM Tris/Cl pH 7.5, 150 mM NaCl, 2 mM PMSF, 1x mammalian protease inhibitor mix M (Serva)) and incubated with 25 μl of the PARP1 nanotrap for 1 h in an end-over-end rotor at 4°C. As a negative control, a non-related nanobody coupled to 4% cross-linked agarose (GFP-Trap or RFP-Trap) were used. The bead pellet was washed two times in 500 μl dilution buffer. After the last washing step, the beads were transferred to a new cup, resuspended in 2x SDS-sample buffer (120 mM Tris/Cl pH 6.8; 20% glycerol; 4% SDS, 0.04% bromophenol blue; 10% β-mercaptoethanol) and boiled for 10 min at 95°C. Samples (1–2% input, 1–2% flow-through, 25–50% bound) were analyzed by SDS-PAGE followed by western blotting.

### SDS-PAGE and immunoblotting

Denaturing polyacrylamid gel electrophoresis (SDS-PAGE) was performed according to standard procedures. Proteins were transferred from SDS gels to nitrocellulose membranes (Bio-Rad) by semi-dry blotting and subsequently probed with different antibodies. Blots were scanned on the Typhoon-Trio laser scanner (GE Healthcare) and quantitatively analyzed with ImageJ software (http://imagej.nih.gov/ij/).

### In vitro synthesis of pADPr polymers

pADPr polymer synthesis was performed according to the protocol from [25], with modifications detailed in [23, 26]. Here, pADPr polymers were synthesized using endogenous hPARP1 immobilized on PARP1 nanotrap and compared with the pADPr polymer synthesis catalyzed by “free” recombinant hPARP1 purified from *E*. *coli*.

### Surface plasmon resonance

Affinity measurements with Biacore T200 (GE-Healthcare) were kindly conducted by PD Dr. Ralf Heermann at the LMU Munich. SPR sensorgrams were subsequently recorded using the Biacore T200 Control software 1.0 and the resulting data was analyzed with the Biacore T200 Evaluation software 1.0. The PARP1 nanobody was captured on a carboxymethyldextran chip (Xantec) via its C-terminal His_6_-tag by immobilizing an anti-His antibody (His Capture Kit 28-9950-56, GE Healthcare) through standard covalent amino-coupling to the chip surface. Recombinantly purified hPARP1 was passed over the chip in seven different concentrations from 10 nM to 1000 nM, with the lowest concentration injected twice as internal control. The hPARP1 injection time was 3 min, followed by a dissociation time of 10 min. The surface was regenerated with 10 mM glycine pH 1.5 for 30 sec followed by a stabilization period of 10 sec. The surface of the flow cell 1 was used to generate blank sensorgrams for substraction of bulk refractive index background. The reference sensorgrams were normalized to a base line of 0. Peaks in the sensorgrams at the beginning and the end of the injection emerged from the run time difference between the flow cells of the chip.

### Cells culture and transfections

HEK293T, HeLa, HT1080, MCF7, U2OS, PC3 and BHK cells were cultivated according to standard protocols. Briefly, growth media consisted of DMEM (high glucose, pyruvate, L-Glutamine) supplemented with 10% fetal calf serum (FCS) and antibiotics. Cells were trypsinized for passaging and cultivated at 37°C in a humidified chamber with a 5% CO2 atmosphere. Plasmid-DNA was transfected with Lipofectamine^®^ 2000 (Life Technologies) according to the manufacturer´s protocol. HeLa cells stably expressing the PARP1 chromobody were generated by transfection of the PARP1 chromobody vector (PARP1 V_H_H fused to TagRFP) and selection of resistant clones with G418 (1 μg/μl) followed by single-clone cell sorting by FACS. For live-cell imaging, the cells were cultivated in DMEM without phenol red and supplemented with 5% FCS and 10 mM sterile HEPES (Sigma).

### Resazurin assay

To test an overall impact of the chromobody on cell viability and metabolic status, untransfected and transfected HeLa cells 16 h post-transfection with the PARP1 chromobody plasmid were incubated for 24 h in cell culture medium containing resazurin (alamarBlue^®^, AbD Serotec). The assay was performed and evaluated according to the manufacturer’s protocol. Absorbance was measured at 570 nm and 600 nm with a spectrophotometer (Multiskan^™^ Go, Thermo Scientific).

### Cell fixation and immunocytochemistry

For end-point analysis, cells were fixed with 3.7% formaldehyde in PBS for 10 min at RT. For detection of pADPr polymers with immunofluorescence, HeLa cells expressing/not expressing PARP1 chromobody grown on glass coverslips, were treated with 10 mM H_2_O_2_ (Sigma) for 10 minutes, fixed with ice-cold methanol/acetone (1:1) and incubated with anti-pADPr antibody (clone 10H, Santa Cruz) followed by secondary antibody. Subsequently, nuclei were stained with DAPI (Invitrogen).

### Fluorescent Two-Hybrid assay (F2H^®^)

Cell-based F2H^®^ protein-protein interaction assay was carried out with F2H Kit Basic (ChromoTek) according to manufacturer’s instructions.

### Microscopy and image analysis

Epifluorescence imaging was performed using a Leica wide-field fluorescence microscope equipped with a 20x objective (Leica). F2H and HCA images were acquired with the InCell Analyzer 1000 (GE Healthcare) from 30 positions per well in an automated fashion. For evaluation of nucleoli, automated image analysis was carried out with an IN Cell Analyzer 1000 Workstation 3.5 (GE Healthcare). “Multi-target analysis” segmentation was performed to segment nuclei (based on their fluorescent intensity, size and shape in the DAPI channel), cells (“collar” in RFP channel) and organelles (nucleoli in the nuclei in RFP channel, based on the size). This allowed identification of morphologically appropriate nuclei, defining cytoplasmic area around the nuclei and nucleoli in the nuclei. Cell-by-cell analysis was performed for at least 100 cells stably expressing PARP1 chromobody per well. Percentage (%) of cells with nucleoli was calculated by normalizing the number of cells with more than one nucleolus to the total number of cells with PARP1 chromobody signal.

### Laser microirradiation

Laser microirradiation live-cell experiments were carried out on an UltraView Vox spinning disc microscope with integrated FRAP PhotoKinesis accessory (Perkin Elmer) assembled to an Axio Observer D1 inverted stand (Zeiss) and using a 63x/1.4 NA Plan-Apochromat oil immersion objective. The microscope was equipped with a heated environmental chamber set to 37°C. Fluorophores were excited with 561 nm solid-state diode laser lines. Confocal image series were recorded with 14-bit image depth, a frame size of 256 × 256 pixels and a pixel size of 110 nm. Microirradiation was carried out with a 405 nm diode laser set to 100% emission. Preselected spots of ~1 μm in diameter within the nucleus were irradiated for 1 s. Before and after microirradiation, confocal image series of one mid z-section were recorded at 1 s time interval (5 or 9 pre-irradiation and 100 or 130 post-irradiation frames). For evaluation of the recruitment kinetics, fluorescence intensities of the irradiated region were corrected for background and normalized to the pre-irradiation values. Data from 10–14 microirradiated cells of each cell type were averaged and plotted.

### Carbon ion beam microirradiation

Carbon ion microirradiation was performed at the Munich ion microbeam SNAKE facility (Supraleitendes Nanoskop für Angewandte Kernphysikalische Experimente, Maier-Leibnitz-Laboratory, Garching, Germany) [27–29]. For irradiation and live-cell imaging, HeLa cells were transfected with the PARP1 chromobody and re-seeded into live-cell imaging cell containers, where cells grow on a BC418 plastic scintillator [28, 30]. Cells were cultivated in phenol red free medium supplemented with 2.5 mM HEPES and 0.25 mM Trolox. Carbon ions of 55 MeV total energy with a LET in water of 310 keV/μm were used in this work (count rate 1.5 Hz). Individual cell nuclei were irradiated with defined numbers of ions (30 or 300) per dot in five-dot irradiation patterns as described [27]. Distance between dots was 3 μm. About ten nuclei were targeted in a single irradiation, which takes about 1 s for 30 ions per dot or about 10 s for 300 ions per dot. Image acquisition was performed with an inverse epifluorescence microscope (Zeiss Axiovert 200M Z1) using a Zeiss Plan Apochromat 40x/0.95 objective (Korr Ph3 M27) and the software AxioVision 4.6 and an AxioCam Mr3 camera. Cell chambers were kept at 37°C during image acquisition.

## Results

### PARP1 nanobody efficiently immunoprecipitates endogenous hPARP1

**Fig 1:**
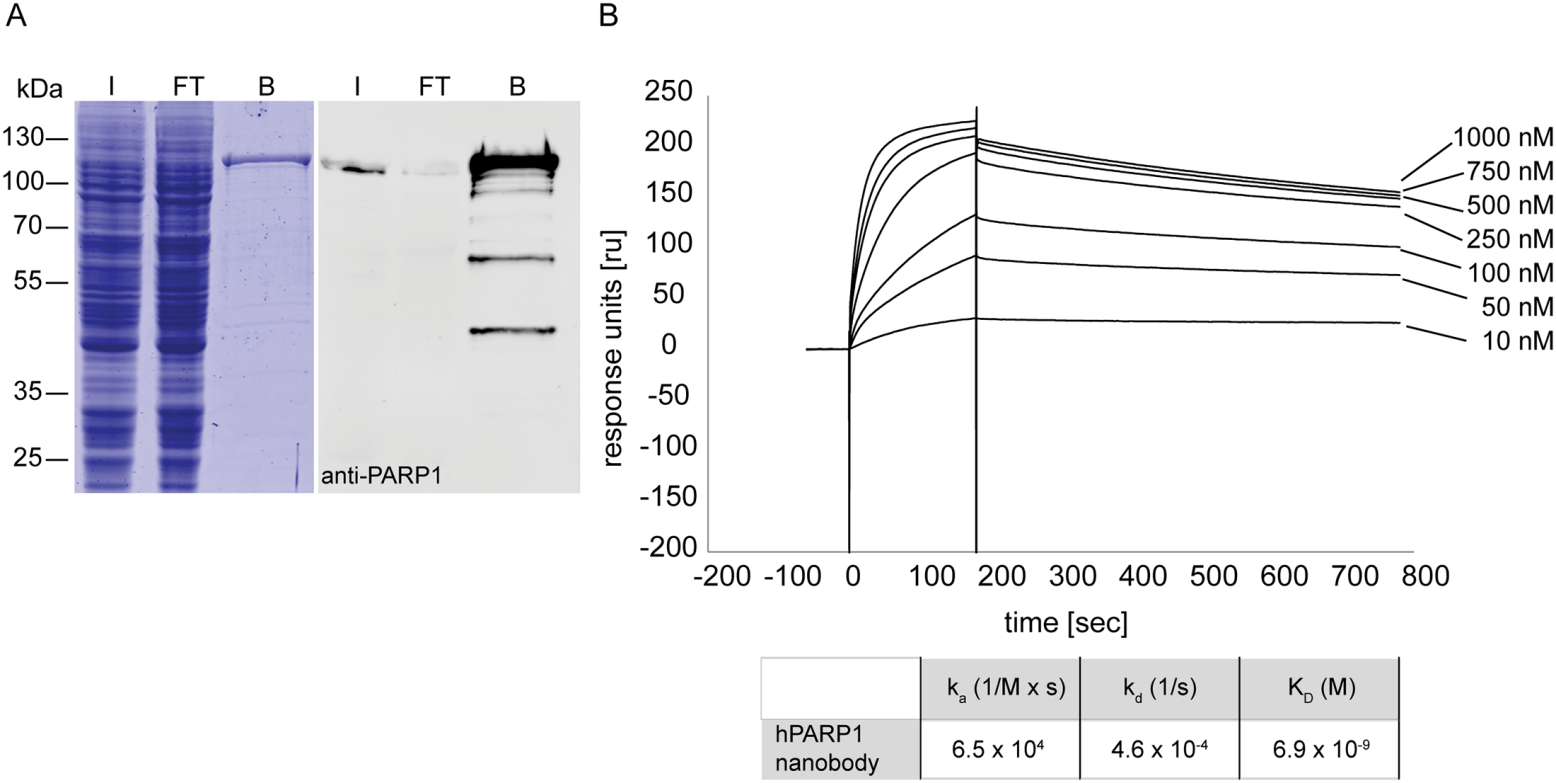
Immunoprecipitation performance and affinity of the PARP1 nanobody. (A) Immunoprecipitation of endogenous hPARP1 from whole-cell lysates of HEK293T cells with PARP1 nanotrap. Input (I), flow-through (FT) and bound (B) fractions were analyzed by SDS-PAGE followed by Coomassie Blue staining (left) and western blotting with anti-PARP1 antibody (right). (B) Affinity measurement of the PARP1 nanobody with Biacore SPR. The sensorgrams for the nanobody at different concentrations of hPARP1 are indicated.

We isolated a nanobody against human PARP1 from an immune alpaca V_H_H library (see Materials and Methods, PARP1 nanobody is available via MTA). In a first approach, we tested the performance of this nanobody to precipitate endogenous PARP1. To this end, we generated an affinity resin, further referred to as PARP1 nanotrap, by covalently coupling the purified nanobody to functionalized agarose beads via internal primary amino groups. We incubated the PARP1 nanotrap with the soluble fraction of a whole-cell lysate of human embryonic kidney cells (HEK293T). Subsequently we analyzed the input, non-bound and bound fractions by SDS-PAGE followed by Coomassie Blue staining or immunoblotting with anti-PARP1 antibody. The results show that PARP1 nanotrap efficiently precipitates endogenous human PARP1 protein with the expected size of ~113 kDa (Fig 1A). The monoclonal anti-PARP1 antibody also detects several smaller degradation bands in the bound lane.

Further we measured the affinity of the purified PARP1 nanobody to the recombinant human PARP1 (hPARP1) using Biacore surface plasmon resonance (SPR) technology (GE Healthcare). After immobilizing the PARP1 nanobody on the chip association/dissociation rates were measured by injecting serial dilutions of seven different concentrations (10 nM– 1000 nM) of recombinant hPARP1. We determined that the PARP1 nanobody affinity (K_D_ value) lies in the low nanomolar range of ~ 6.9 nM (Fig 1B), which correlates with the high immunoprecipitation ability of the PARP1 nanotrap observed in the pull-down experiments.

### PARP1 nanobody is restricted in selectivity across the PARP family and in species reactivity

**Fig 2:**
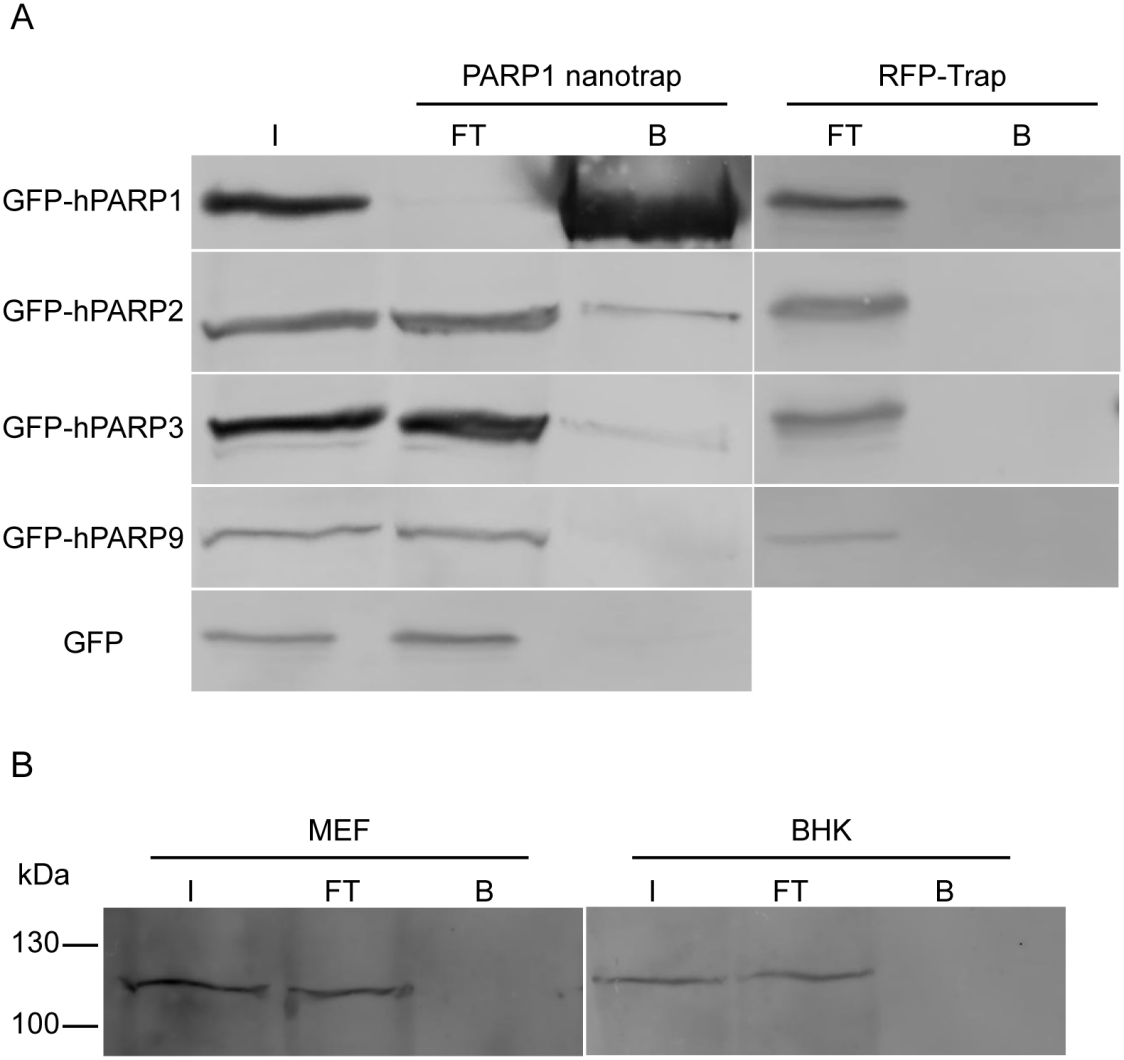
Determination of the PARP family selectivity and species reactivity of the PARP1 nanobody. (A) Immunoprecipitation of GFP-tagged hPARP1 (141 kDa), hPARP2 (93 kDa), hPARP3 (87 kDa), hPARP9 (123 kDa) and GFP (27 kDa, negative control) with the PARP1 nanotrap from transiently transfected HEK293T cells. RFP-Trap was used as control. Input (I), flow-through (FT) and bound (B) fractions were separated by SDS-PAGE followed by immunoblotting with anti-GFP antibody. (B) Immunoprecipitation of endogenous PARP1 from mouse (MEF) and hamster (BHK) cells with the PARP1 nanotrap. The fractions were analyzed by SDS-PAGE and immunoblotting with anti-PARP1 antibody.

The human PARP proteins share a conserved catalytic domain, the so-called PARP-signature region. Hence, we asked whether PARP1 nanobody recognizes other abundant members of the PARP family. For this, GFP-fusions of hPARP1, hPARP2, hPARP3 and hPARP9 were expressed in HEK293T cells and subjected to pull-down experiments using the PARP1 nanotrap (Fig 2A). The analysis shows that the PARP1 nanotrap specifically precipitates GFP-hPARP1, whereas no binding of GFP-hPARP9 is detectable. Regarding the weak signal in the bound fraction of GFP-hPARP2 and GFP-hPARP3 we speculate that the slight pull-down of GFP-hPARP2 and GFP-hPARP3 results rather from co-immunoprecipitation of hPARP2 and hPARP3 with endogenous hPARP1 than from direct binding of the PARP1 nanotrap to hPARP2 and hPARP3 (Fig 2A). This speculation is supported by previous observations describing PARP1 to interact with PARP2 [31, 32] and hPARP3 [33, 34]. Since the PARP1 enzyme is characterized by a relatively high sequence homology across different mammalian species, we examined cross-species reactivity of the PARP1 nanobody. To this end, we performed immunoprecipitations incubating the PARP1 nanotrap with soluble protein fractions of whole-cell lysates either derived from human embryonic kidney cells (HEK293T), mouse embryonic fibroblasts (MEF) or baby hamster kidney fibroblasts (BHK). Immunoblot analyses of the bound fractions reveals that the PARP1 nanotrap exclusively recognizes human PARP1 (shown in Fig 1A for endogenous hPARP1 and in Fig 2A for overexpressed GFP-hPARP1), but neither mouse nor hamster PARP1 (Fig 2B).

### The epitope of the PARP1 nanobody is localized within the DNA-binding domain of hPARP1

**Fig 3:**
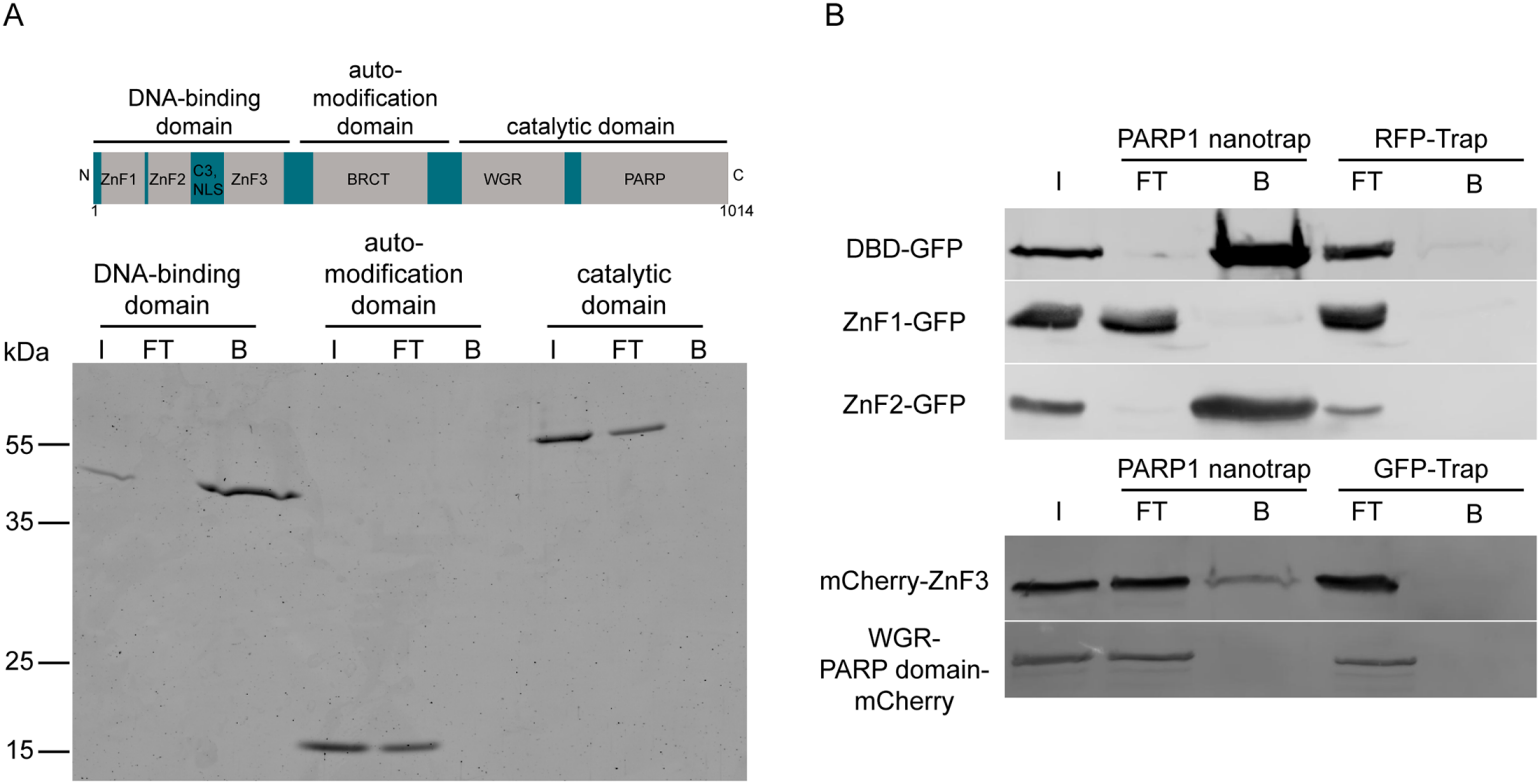
Epitope mapping of the PARP1 nanobody by immunoprecipitation of hPARP1 domains. (A) Schematics depicts hPARP1 domain structure: DNA-binding domain (45 kDa), automodification domain (19 kDa) and catalytic domain (58 kDa). Purified recombinant hPARP1 domains were subjected to immunoprecipitation with the PARP1 nanotrap Input (I), flow through (FT) and bound (B) fractions were analyzed by SDS-PAGE followed by Coomassie Blue staining. (B) GFP- or mCherry-tagged hPARP1 domains were transiently expressed in HEK293T cells: full DNA-binding domain (DBD), DBD constituting zinc fingers (ZnF1, ZnF2, ZnF3), as well as the WGR domain (part of the catalytic domain). The cells were lysed and immunoprecipitated with the PARP1 nanotrap and RFP-Trap or GFP-Trap as control. The fractions were subjected to SDS-PAGE followed by immunoblotting with anti-GFP or anti-RFP antibody.

To narrow down the binding region of the PARP1 nanobody, the hPARP1 protein was genetically fragmented into three major domains: a DNA-binding domain (DBD), an automodification domain and a catalytic domain. The individual domains were expressed in SF9 insect cells, purified and tested for binding to the PARP1 nanotrap in pull-down experiments. Immunoprecipitation revealed that only the DBD of hPARP1 was recognized by the PARP1 nanotrap (Fig 3A) whereas no binding of the automodification or catalytic domain was detected.

In a next step, we analyzed binding of the PARP1 nanotrap to the three individual zinc finger domains (ZnF1, ZnF2 or ZnF3) of the DBD of hPARP1. We showed that the PARP1 nanotrap preferentially captures the zinc finger 2 region (ZnF2) of the DBD (Fig 3B). Whereas no binding to ZnF1 or the WGR domain (part of the catalytic domain, served as negative control) was observed, we detect a weak binding to ZnF3. This could be a hint that the PARP1 nanobody recognizes an extended three-dimensional epitope within the DBD.

These findings indicate that the epitope of the PARP1 nanobody is predominantly localized within the ZnF2 region of the DNA-binding domain of human PARP1. Most interestingly, sequence alignments of ZnF2 between human, mouse or hamster revealed only three positions with different amino acid residues (positions 161, 188, and 189) (S1A Fig). To test whether these residues form an essential part of the nanobody epitope, we performed site-directed mutagenesis turning the human ZnF2 into a hamster/mouse ZnF2. Subsequently we tested the mutated ZnF2 in pull-down experiments with the PARP1 nanotrap (S1B Fig). Analysis of the bound fractions showed that the mutation of the three selected amino acids within the human ZnF2 resulted in an approximately seven fold decrease of the binding to the PARP1 nanotrap (S1C Fig). This data suggests that the amino acids 161, 188, and 189 within the ZnF2 are involved in the epitope recognition by the PARP1 nanobody.

### Nanobody-bound hPARP1 retains its enzymatic activity

To test the effect of nanobody binding on the catalytic activity of PARP1, we developed an *in vitro* on-bead poly(ADP-ribosyl)ation assay. We used the PARP1 nanotrap for one-step pull-down of endogenous hPARP1 from HEK293T cells. Subsequently, the bound protein was directly used for *in vitro* poly(ADP-ribosyl)ation followed by pADPr chain extraction. After extraction, the protein-free pADPr chains were analyzed for UV-absorbance and subjected to 20% native gel electrophoresis followed by silver staining.

**Fig 4:**
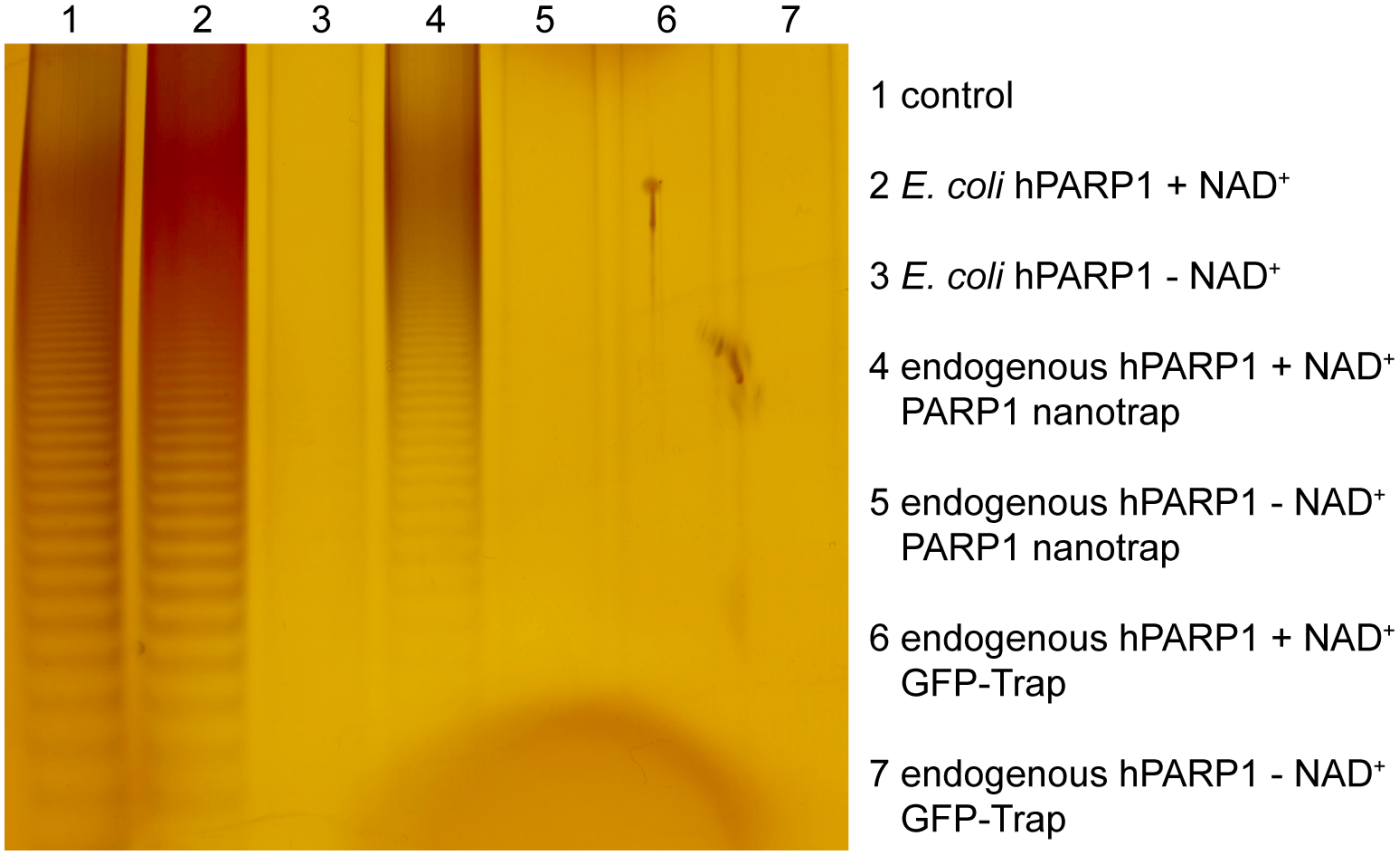
On-bead pADPr chain synthesis with the endogenous hPARP1 immunoprecipitated with the PARP1 nanotrap. Gel electrophoresis and silver staining of pADPr fractions from *in vitro* synthesis. Lanes 1–7: commercially available pADPr chains (lane 1, control); reaction with the purified recombinant hPARP1 with NAD+ (lane 2) or without NAD+ (lane 3); on-bead reaction with PARP1 nanotrap-precipitated endogenous hPARP1 with NAD+ (lane 4) or without NAD+ (lane 5); on-bead reaction with GFP-Trap with NAD+ (lane 6) or without NAD+ (lane 7).

Both, absorbance spectra (S2 Fig) and gel electrophoresis (Fig 4) showed that the endogenous hPARP1 bound to the nanotrap was able to synthesize pADPr polymers. We detected a comparable pADPr chain pattern in the reactions performed with the nanotrap-bound endogenous hPARP1 as well as with the purified recombinant hPARP1 used as a positive control. No pADPr chains were detected in the internal control samples without NAD+ as substrate or in samples obtained with an unrelated nanotrap (GFP-Trap). These results indicate that the nanobody-bound hPARP1 retains its enzymatic activity.

### PARP1 chromobody specifically recognizes hPARP1 in live cells

Next, we asked whether the PARP1 nanobody is able to recognize its target not only *in vitro*, but also in its native cellular environment. To detect endogenous PARP1 within living cells, we fused the coding sequence of the nanobody to the red fluorescent protein TagRFP, generating a so-called PARP1 chromobody. Upon intracellular expression, the PARP1 chromobody becomes visible and can be analyzed using fluorescence microscopy.

**Fig 5:**
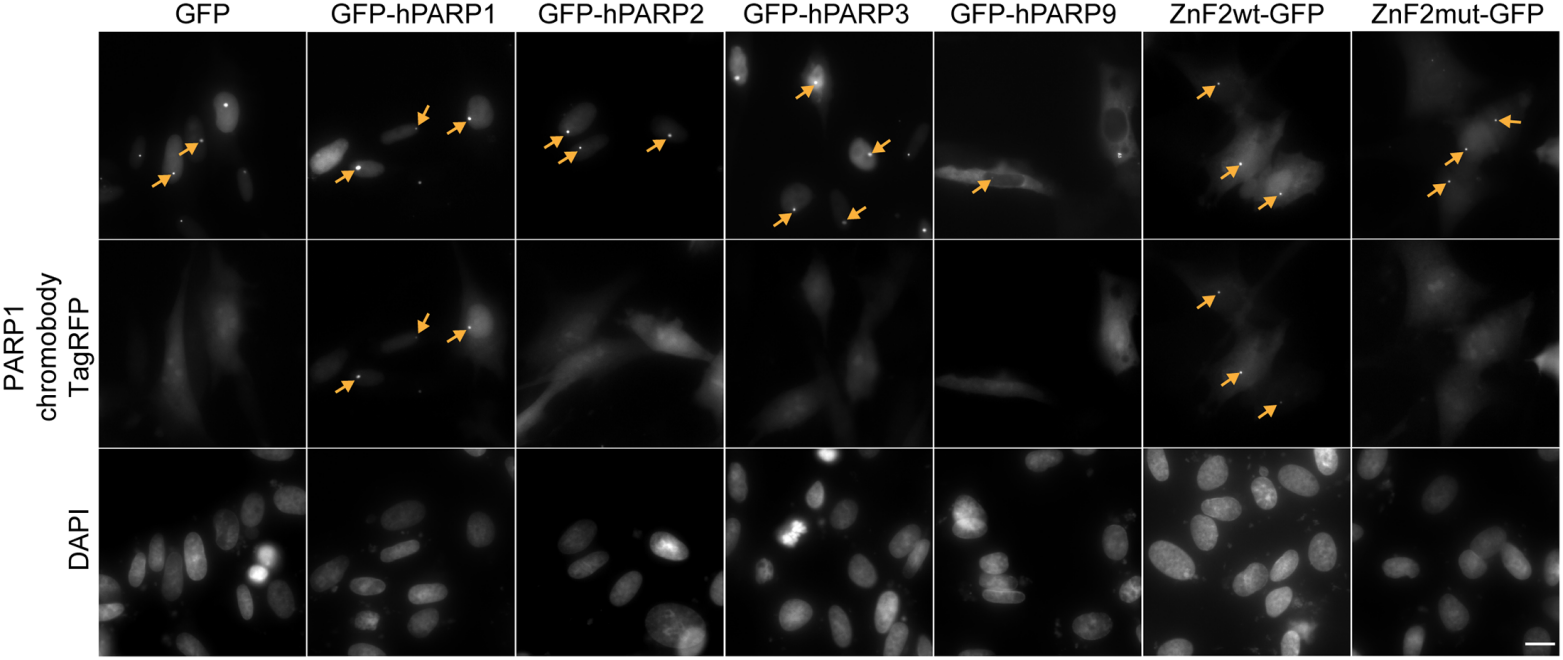
Intracellular F2H analysis of the PARP1 chromobody. BHK-F2H cells were pairwise co-transfected with the PARP1 chromobody fused to TagRFP and one of the GFP-tagged bait constructs: GFP alone, GFP-hPARP1, GFP-hPARP2, GFP-hPARP3, GFP-hPARP9, wild-type ZnF2-GFP and ZnF2mut-GFP. The cells were fixed, stained with DAPI and subjected to fluorescence microscopy. Upper row, green channel: GFP-fusion proteins are enriched at the “spot” in the nuclei of transfected BHK-F2H cells (arrows). Middle row, red channel: binding of the PARP1 chromobody to the full-length GFP-hPARP1 and to the wild-type ZnF2-GFP is visible as local enrichments of the red fluorescent signals (arrows). Neither interaction of the PARP1 chromobody with hPARP2, 3, or 9, nor interaction with the mutant ZnF2mut-GFP construct (G161T, A188S and T189A) can be observed. Co-transfection with GFP (first column) served as negative control to exclude non-specific binding of the PARP1 chromobody to GFP. Scale bar, 5 μm.

In a first step, we investigated whether the PARP1 chromobody recognizes hPARP1 intracellularly. To this end, we performed a Fluorescent Two-Hybrid (F2H) assay [35, 36]. This live-cell protein-protein interaction assay relies on a tethering strategy, where a GFP-fused bait is immobilized at a particular protein interaction platform (“spot”) in the nucleus of genetically engineered BHK cells (F2H-BHK). To analyze the chromobody binding, we co-transfected different GFP-tagged bait proteins and the PARP1 chromobody pairwise into these F2H-BHK cells. Here, different GFP-hPARP fusion proteins served as a bait, whereas the PARP1 chromobody fused to TagRFP served as a prey. The cells co-expressing both bait and prey were evaluated for co-localization of green (GFP-tagged constructs) and red (chromobody) fluorescent signals by microscopy. Image analysis revealed that the PARP1 chromobody co-localized with GFP-hPARP1, which was enriched at the “spot” in BHK-F2H cells (Fig 5). When GFP alone was enriched at the “spot”, the PARP1 chromobody showed no binding, but a disperse distribution within the nucleus and, to a lesser extent, in the cytoplasm. In accordance with our biochemical findings, the F2H^®^ assay revealed no binding of the PARP1 chromobody to GFP-fusions of hPARP2, hPARP3 or hPARP9. Furthermore, the PARP1 chromobody recognized the GFP-tagged ZnF2 domain but not the mutated ZnF2-GFP (G161T, A188S and T189A). These findings indicate that PARP1 chromobody is functional upon expression within living cells.

**Fig 6:**
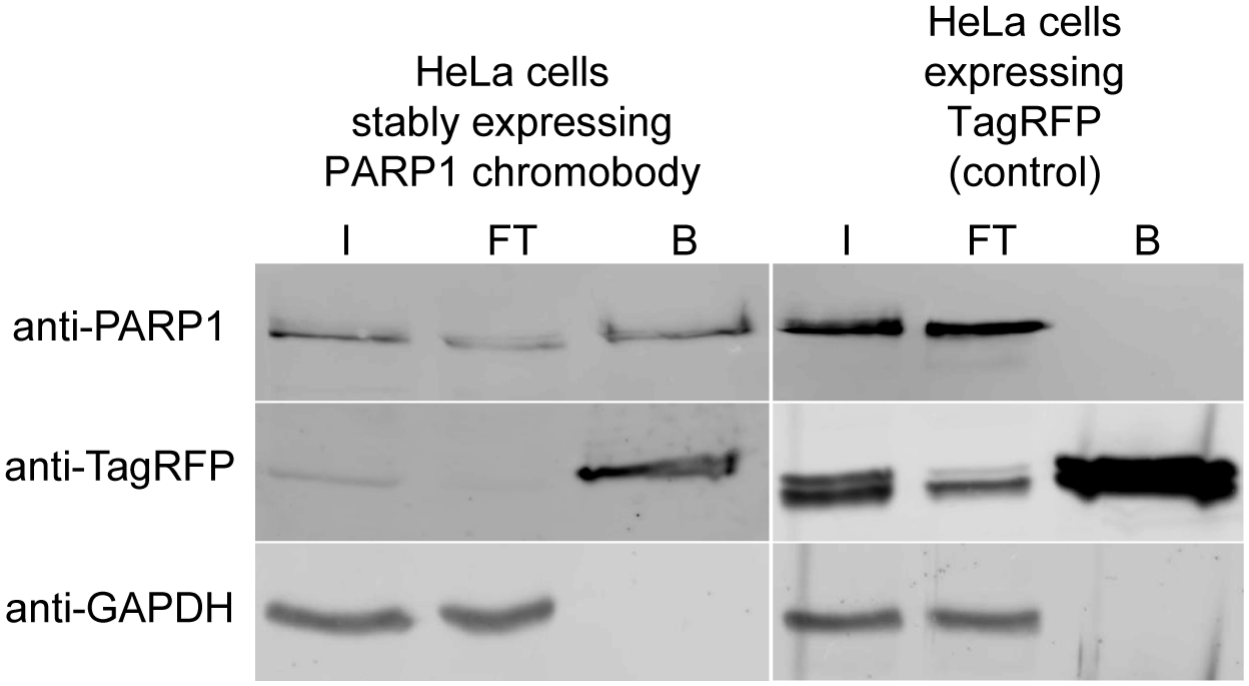
Endogenous hPARP1 co-precipitates together with the intracellularly expressed PARP1 chromobody. PARP1 chromobody fused to TagRFP was precipitated using the RFP-affinity resin (RFP-Trap) from a whole-cell lysate of HeLa cells stably expressing the chromobody. TagRFP-transfected HeLa cells served as negative control for non-specific binding. Input (I), flow-through (FT) and bound (B) fractions were subjected to SDS-PAGE and immunoblotting with anti-PARP1 antibody, followed by anti-TagRFP antibody and anti-GAPDH antibody as loading control.

In addition, we tested intracellular binding properties of the PARP1 chromobody biochemically by performing intracellular immunoprecipitation [17]. We used the TagRFP-tag of the PARP1 chromobody as an affinity tag for pull-downs with the RFP-affinity resin (RFP-Trap). We prepared soluble protein fractions of whole-cell lysates derived from HeLa cells stably expressing PARP1 chromobody (fused to TagRFP). As a negative control, we used HeLa cells expressing only TagRFP. Subsequently, we incubated the soluble protein fractions with the RFP-Trap to precipitate the PARP1 chromobody in complex with endogenous PARP1 and analyzed the bound fraction by immunoblotting using antibodies specific for TagRFP and PARP1 (Fig 6). The data showed that the PARP1 chromobody and the TagRFP protein were highly enriched in the bound fraction. In addition, endogenous hPARP1 was co-immunoprecipitated with the PARP1 chromobody but not with the TagRFP alone. From these results we conclude that the PARP1 chromobody specifically binds endogenous PARP1 upon intracellular expression.

### PARP1 chromobody enables dynamic visualization of the endogenous PARP1 localization in live human cells

**Fig 7:**
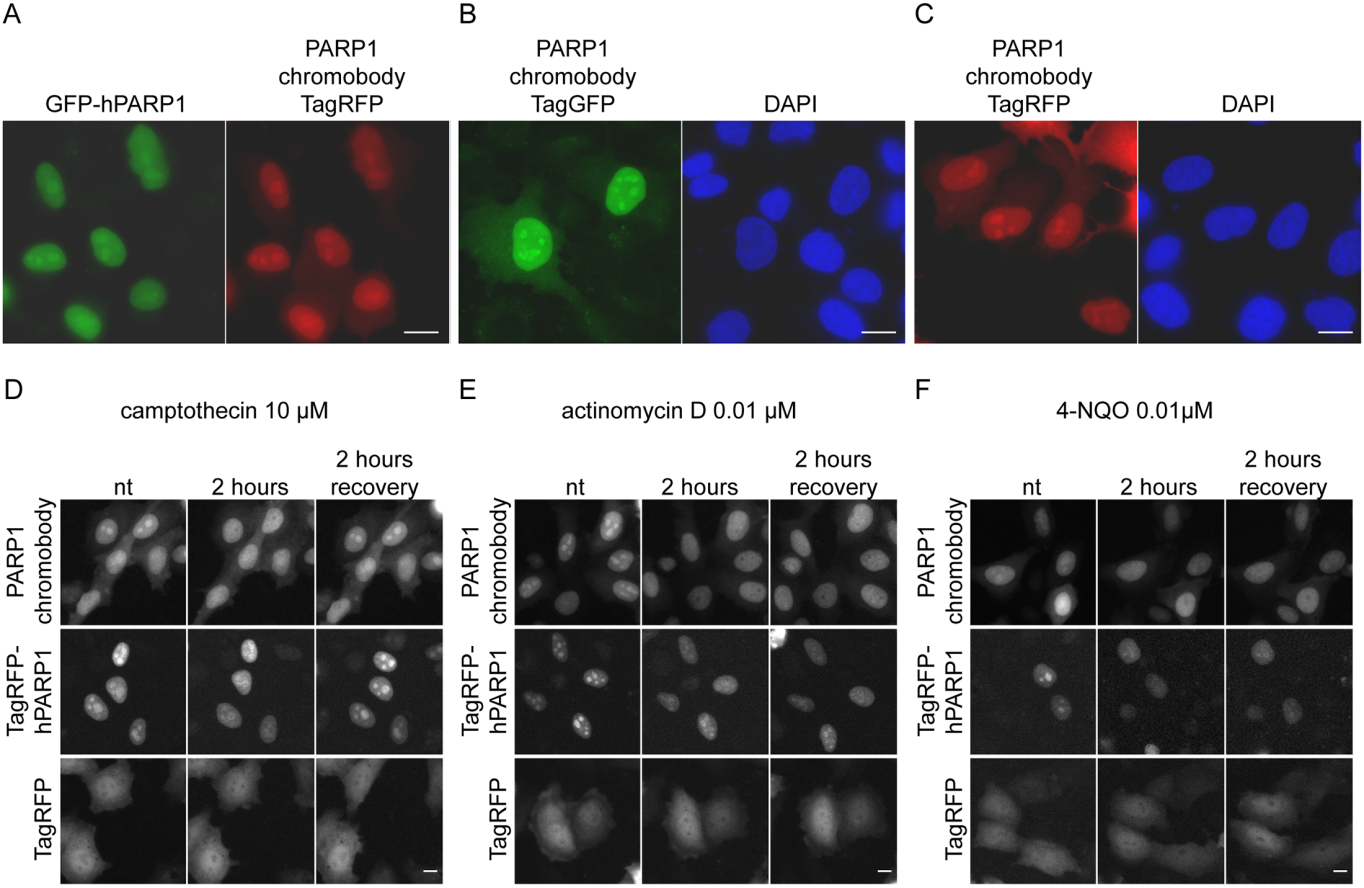
PARP1 chromobody enables visualization of hPARP1 in human HeLa cells. (A) PARP1 chromobody fused to TagRFP (red) co-localizes with GFP-PARP1 (green) in nucleoli and nucleoplasm. (B-C) PARP1 chromobody fused to TagGFP (B, green) or fused to TagRFP (C, red) visualizes endogenous hPARP1 in nucleoli and nucleoplasm. Cells were fixed, stained with DAPI (blue) and subjected to epifluorescence imaging. Scale bar, 10 μm. (D-F) Live-cell imaging with the PARP1 chromobody upon compound treatment. HeLa cells transiently expressing either PARP1 chromobody, TagRFP-hPARP1 or TagRFP alone were treated for 2 h with 10 μM camptothecin (D), 0.01 μM actinomycin D (E), or 0.01 μM 4-NQO (F). After subjecting cells to incubation with the compounds, cells were washed and allowed to recover for another 2 h. Time-lapse epifluorescence imaging was carried out in an automated fashion every 15 min during treatments and during recovery. The panels show selected frames of the cells before treatments, after 2 h of treatment and after 2 h of recovery. Scale bar, 5 μm.

To determine whether the intracellular expression of the hPARP1 chromobody permits visualization of the endogenous hPARP1, we further analyzed subcellular localization of the PARP1 chromobody. When co-transfected in HeLa cells, the PARP1 chromobody co-localizes with the GFP-tagged hPARP1 in the nucleoplasm and nucleoli (Fig 7A). Similarly, when transfected alone, the PARP1 chromobody was localized predominantly to the nucleus with enrichment in the nucleoli (Fig 7B and 7C), recapitulating localization of the endogenous hPARP1 described in the literature [37–39]. This observation suggests that the chromobody is recruited to endogenous hPARP1 and does not bind non-specifically to other cellular structures. The cytoplasmic background of the chromobody might be due to unbound chromobody upon overexpression. Indeed, HeLa cells stably expressing lower levels of PARP1 chromobody almost totally lacked the chromobody signal in the cytoplasm (data not shown).

Whereas in all tested human cell lines (HeLa, HT1080, MCF7, U2OS, HEK293T, PC3) the chromobody signal was localized in the nuclei and nucleoli, in non-human cell lines (mouse MEFs or hamster BHK) the chromobody signal was dispersed in the cytoplasm and, to a lesser extent, in the nucleus (S5 Fig, left column). This data correlates well with our biochemical analysis indicating the specificity of the chromobody to human PARP1, but not to mouse or hamster PARP1.

We further asked whether the PARP1 chromobody can be applied to trace the dynamics of PARP1 subnuclear localization. It has been shown in the past, that treatment of cells with rRNA transcription inhibitors or DNA damaging agents induces PARP1 delocalization from nucleolus to nucleoplasm [13–15, 40]. However, those results were based on immunofluorescence studies or overexpression of fluorescently labeled PARP1. Therefore, we sought to visualize this translocation at endogenous level using the PARP1 chromobody. To monitor delocalization of the endogenous, nucleolar hPARP1 in living cells, HeLa cells transiently expressing the PARP1 chromobody were incubated with the following rRNA transcription inhibitors: camptothecin [41, 42], actinomycin D [43–46] and 4-NQO [47, 48]. Cells overexpressing TagRFP-hPARP1 or TagRFP alone were used as positive and negative controls respectively. Real-time image acquisition was performed during the 2 h incubation, followed by additional 2 h after changing to compound-free medium. The results show a clear alteration of the nucleolar chromobody signal upon treatment with camptothecin, actinomycin D or 4-NQO (Fig 7D–7F, also S1 Video). The signal in the nucleoli of the cells expressing fluorescently labeled hPARP1 (TagRFP-hPARP1) was also decreased. However, a slight nucleolar pattern of TagRFP-hPARP1 was still detectable after 2 h of treatment, which could be explained by the non-physiologically high expression level of the TagRFP-hPARP1. Upon 2 h of recovery, the nucleolar pattern of both chromobody and TagRFP-hPARP1 was completely restored in camptothecin treated cells, but not in actinomycin D and 4-NQO treated cells (Fig 7D–7F, also S1 Video). This data correlates well with the described mode of action of these compounds: camptothecin inhibits processing of ribosomal precursor RNA and it’s action is rapidly reversible [49], whereas actinomycin D-treated cells need a 24 h period to recover their ability to synthesize RNA [50]. In addition to inhibiting rRNA-synthesis, 4-NQO induces DNA damage, and it has been shown previously, that a 24 h recovery period is needed to repair 70% of the DNA damage [51]. No signal alteration was observed in the TagRFP-expressing control cells.

In order to be suitable for live-cell imaging and target monitoring, the chromobody’s influence on the target function and cell viability should be negligible. With a resazurin assay, we could show that the chromobody does not have any significant impact on cell viability and metabolic status (S3 Fig). Further, we demonstrated that the intracellular PARP1 chromobody expression does not detectably affect the enzymatic activity of the endogenous hPARP1. The pADPr-antibody staining of the H_2_O_2_-treated cells shows a typical dotted nuclear pattern both in PARP1-chromobody expressing HeLa cells, as well as in HeLa cells without the chromobody (S3 Fig), which correlates well with our biochemical data. No pADPr pattern could be detected in the untreated cells (control).

Next, we tested the PARP1 chromobody for visualization of redistribution of endogenous hPARP1 in automated compound profiling. For this, HeLa cells stably expressing PARP1 chromobody at a uniform level were seeded in 96-well plates and treated with different concentrations of 4-NQO, actinomycin D, camptothecin and H_2_O_2_ for 4 h (S4 Fig). After the treatment, the cells were fixed, stained with DAPI, and subjected to automated high content imaging determining the percentage of cells with PARP1 in nucleoli before (0 μM) and after (0.01 μM– 1 mM) treatment. Quantitative analysis revealed that 4-NQO and actinomycin D are very potent at very low doses of 0.01 μM, at which they deplete the chromobody signal from the nucleoli. Camptothecin is increasingly effective starting with the dose 0.01 μM up to 100 μM. Treatment with H_2_O_2_ affected the nucleolar signal only when applied in toxic amounts (100 μM and 1 mM). This demonstrates that the PARP1 chromobody enables detailed analysis and comparison of potencies of chemical compounds to redistribute endogenous PARP1 in live human cells.

### PARP1 chromobody visualizes recruitment of hPARP1 to DNA lesions

**Fig 8:**
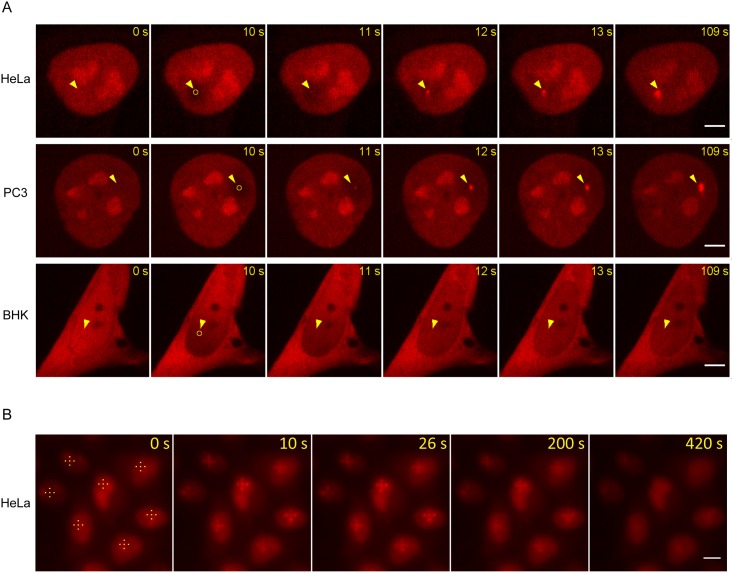
Recruitment of endogenous PARP1 to the DNA damage sites as visualized by the PARP1 chromobody in live human cells. (A) Live-cell imaging of laser-microirradiated (405 nm laser, 100% power, 1 s) human HeLa, human PC3 cells and hamster BHK cells transiently expressing PARP1 chromobody. Time-lapse imaging was carried out at 1 frame per second with the spinning disc microscope acquiring 9 pre-irradiation and 100 post-irradiation frames. Selected time-frames are shown, yellow circles depict the regions of microirradiation (Ø 1 μm), yellow arrow-heads mark the sites before and after irradiation. Scale bar, 5 μm. (B) Live-cell imaging of carbon ion-irradiated HeLa cells (300 ions per point) transiently expressing PARP1 chromobody. The cells were irradiated with accelerated 55 MeV (total energy) carbon ions (LET in water: 310 KeV/μm). At 0 s yellow dots in a cross-shape mark the prospective sites of irradiation. After irradiation images were acquired every ~4 s. Selected time points are shown. Scale bar, 10 μm.

Finally, we analyzed whether the PARP1 chromobody visualizes the recruitment of the endogenous hPARP1 on DNA lesions upon microirradiation. Firstly, we applied an UV laser, which mainly provokes a photochemical reaction through absorption of the transferred energy that induces various DNA lesions including single and double-strand breaks [52, 53]. To visualize recruitment of the endogenous hPARP1 to DNA damage sites, we transiently expressed the PARP1 chromobody in different human cell lines: HeLa, HT1080, MCF7, U2OS, PC3, and HEK293T. Upon expression of the chromobody, the cells were subjected to irradiation using a focused 405 nm UV laser to induce DNA damage sites at preselected spots which a defined size (1 μM) within the nuclei. Before and after microirradiation, confocal image series of one mid z-section were recorded at 1 s time interval with 9 pre-irradiation and 100 post-irradiation frames. Upon microirradiation, the PARP1 chromobody visualizes a rapid recruitment and accumulation of the endogenous hPARP1 protein at the DNA damage sites in all tested human cell lines (selected cell lines shown in Fig 8A, S2 Video, the complete panel is shown in S5 Fig). Overexpression of GFP-hPARP1 was used as a positive control for visualization of the DNA lesions and displayed similar recruitment kinetics as the chromobody (S6 Fig).

We further tested the PARP1 chromobody for visualization of PARP1 recruitment to DNA double-strand breaks induced by carbon ion microirradiation at the Munich microprobe SNAKE [28, 29]. The advantage of the ion microirradiation is that it creates DNA double-strand breaks in quantitatively predictably way [54–56], whereas the UV-laser irradiation induces an artificial composition of various types of poorly characterized DNA damage [56]. To semi-quantitatively estimate the minimum damage load necessary for visible PARP1 accumulation, we took advantage of the highly focused beam, which can deliver a pre-defined number of ions on a spot of about 1 μm diameter [27, 30]. In these experiments, HeLa cells transiently expressing the PARP1 chromobody were subjected to carbon ion irradiation with 30 and 300 ions per point (corresponding to about 315 or 3150 double-strand breaks per point, respectively) [27] at preselected spots. After irradiation, a rapid accumulation of the PARP1 chromobody at DNA damage sites can be visualized which reflects the recruitment of endogenous PARP1. The cross-like irradiation pattern can be detected after ~7–10 s in cells irradiated with 300 ions per point (Fig 8B, S3 Video), while no recruitment of endogenous hPARP1 was observed in cells irradiated with 30 ions or less per point (data not shown). The spots represent the accumulated, endogenous hPARP1 at the sites of damaged chromatin. The signal of the PARP1 chromobody is only transiently present at the DNA repair sites. After gradually fading out it is hardly detectable after 420 s.

## Discussion

Here we describe a new biosensor based on a single-domain antibody derived from an alpaca immune library that allows detection and dynamic tracing of one main component of the DNA repair machinery—human PARP1 –in biochemical assays and living cells. In this study we performed a detailed characterization of the binding properties of the PARP1 nanobody which is essential for intended downstream applications in proteomics or live-cell imaging. We could demonstrate that the PARP1 nanobody specifically recognizes and binds the human PARP1 protein. Since nanobodies have only three hypervariable regions (CDRs), compared to antibody formats derived from conventional IgGs, these binders preferably recognize and bind conformational epitopes e.g. formed by enzymatic pockets of regulatory domains [57, 58]. Consequently, binding of nanobodies often interferes with the function of the targeted antigen [59–61]. Epitope mapping of the PARP1 nanobody revealed a binding site within the zinc finger 2 of the DNA-binding domain of hPARP1. Comparison of ZnF2 domains of mouse or hamster PARP1 proteins, which are not recognized by the PARP1 nanobody, gave rise to the assumption that amino acids Gly161, Glu188 and Thr189 are essential for forming a conformational epitope. ZnF2, together with ZnF1, appears to be involved in binding to strand breaks, albeit not in PARP1 activation upon binding to damaged DNA [6]. Still, binding of the nanobody seems not to affect binding of hPARP1 to DNA as shown by efficient recruitment of the PARP1 chromobody to DNA lesions (Fig 8, S5 and S6 Figs, S2 and S3 Videos). In addition, we demonstrate that binding of the PARP1 nanobody does not affect the PARylation activity of hPARP1 neither *in vitro*, nor *in vivo*. Our biochemical PARylation assays showed that PARP1 retains its catalytic activity when bound by the PARP1 nanotrap (affinity matrix from PARP1 nanobody immobilized on agarose beads) (Fig 4, S2 Fig) and cellular immunocytochemistry revealed that PARylation takes place upon H_2_O_2_ treatment of cell expressing the PARP1 chromobody (S3 Fig).

The sensitivity of the PARP1 nanobody further relies on its high affinity (K_D_ ~6.9 nM for hPARP1). In comparison to other recently reported nanobodies, such as the p53 nanobody, which shows a 100 fold lower affinity (K_D_ ~1 μM) [59], the PARP1 nanobody belongs to the group of high-affinity binders. For proteomic approaches we tested the PARP1 nanobody as an affinity reagent. We generated a PARP1 nanotrap by covalently coupling the monovalent PARP1 nanobody to an immobilizing matrix and demonstrated that it enables a fast and efficient one-step immunoprecipitation of the hPARP1 protein from human cell lysate (Fig 1). Such affinity purification reagents are highly favorable due to the properties of the nanobodies regarding stability, chemical resistance, long shelf-life and robust binding performance [24, 62].

In addition, our data shows that the nanobody retains its binding specificities upon intracellular expression (Figs 5 and 6). Until now, for investigation of the subcellular PARP1 localization, the two main techniques were available: immunostaining and ectopic expression of fluorescently tagged PARP1. However, artificially introduced ectopic fusion proteins can lead to phenotypical changes, i.e. the chimeric fusion protein can behave differently than its endogenous equivalent [20, 63]. Furthermore, when a vector-based rather than a knock-in strategy is chosen, transfected cells already express the endogenous variant of the protein and the ectopic expression changes the intracellular amount of available protein. The major drawback of the immunostaining approach is that in order to label an intracellular target, cells have to be fixed and permeabilized, which is not compatible with live-cell analysis and can introduce artifacts and aberrations [11, 12]. A recent study evaluating subcellular localization of over 500 human proteins with immunofluorescence *vs*. chimeric overexpression revealed frequent discrepancies between the data [12]. Therefore, we asked if the PARP1 nanobody could overcome these technical limitations and provide information about localization of the endogenous hPARP1 in live cells without fixation and permeabilization. Upon expression of fluorescent protein-tagged constructs of the PARP1 nanobody (PARP1 chromobody) in live mammalian cells, the chromobody visualizes endogenous hPARP in its native intracellular environment.

In accordance to previous studies using immunofluorescence or overexpression of fluorescently labeled PARP1 [37, 64–66], the PARP1 chromobody signal showed a clear enrichment in the nuclei and nucleoli, which is characteristic for PARP1. In extensive time-lapse analyses we monitored translocation of the chromobody signal from nucleoli into nucleoplasm upon treating cells with inhibitors of rRNA transcription (Fig 7D–7F).

We also showed that the PARP1 chromobody signal translocation can be imaged and quantitatively analyzed in a high content imaging approach (S4 Fig). This approach could enable screening and HCA of PARP1-targeting compounds with respect to their effect on the subnuclear localization of the endogenous PARP1 in live human cells. For example, drug-induced trapping of endogenous PARP1 at DNA lesions could be addressed directly in live human cells with the help of PARP1 chromobody.

We further demonstrate that upon DNA damage induction by microirradiation, the PARP1 chromobody visualizes recruitment of endogenous hPARP1 enzyme to damaged DNA. These experiments revealed that the endogenous hPARP1 is recruited to damaged DNA within seconds, as we observed rapid local enrichment of the chromobody signal at the lesions after a few seconds post-irradiation (Fig 8, S5 Fig, S2 and S3 Videos). The kinetics of hPARP1 recruitment monitored by the chromobody is in line with the previous studies where GFP-PARP1 recruitment was analyzed [67]. Also, previous reports indicate that association of PARP1 with DNA strand breaks is transient [5, 67], and during this study we could show that the enriched chromobody signal at the mircoirradiation sites fades within minutes after irradiation (Fig 8B). By localized carbon ion irradiation we also observed that the local density of DNA lesions has to be very high to enable detection of hPARP1 accumulation.

It is important to note that although the chromobody enables visualization of hPARP1 recruitment, it does not provide information about PARP1 enzymatic activity. For example, upon treatment with H_2_O_2_, no pADPr-like pattern representing active sites of DNA damage could be observed neither with the PARP1 chromobody, nor with TagRFP-hPARP1, but only with a pADPr-specific antibody. This is in agreement with the previous studies showing that even catalytically inactive PARP1 mutants are still recruited to DNA lesions [67].

## Conclusions

In conclusion, here we characterized a novel PARP1 nanobody derived from an alpaca heavy-chain only antibody. We showed that the nanobody is highly affine and specific to the human PARP1 protein. In biochemical applications, PARP1 nanobody enables efficient immunoprecipitation of endogenous human PARP1 and its interaction partners. Furthermore, binding of the nanobody does not disturb enzymatic activity of hPARP1 or its DNA-binding ability. In live-cell experiments we showed that the PARP1 chromobody enables previously impossible real-time visualization of the endogenous hPARP1 enzyme. For the first time, by use of the PARP1 chromobody, the recruitment of the endogenous hPARP1 to the sites of DNA damage could be observed in live cells. Being a versatile affinity reagent functional both *in vitro* and *in vivo*, the newly developed PARP1 nanobody will contribute to further understanding of the various PARP1 functions including the molecular role of PARP1 in DNA repair.

## Supporting Information

S1 Fig Amino acid substitutions within the human PARP1 ZnF2 domain significantly decrease binding of the PARP1 nanotrap. (A) Amino-acid sequence alignment of the PARP1 ZnF2 domains from hamster (*Mesocricetus auratus*, NCBI Reference Sequence: XP_005078195.1), mouse (*Mus musculus*, NCBI Reference Sequence: NP_031441.2) and human (*Homo sapiens*, NCBI Reference Sequence: NP_001609.2). Differences with respect to the human amino acid sequence are highlighted in yellow. Positions for the introduced single mutations are highlighted in green. (B) HEK293T cells were transiently transfected with the GFP-tagged wild-type or mutated (G161T, A188S and T189A) ZnF2 domain of hPARP1, lysed and subjected to pull-down with the PARP1 nanotrap. Input (I), flow-through (FT) and bound (B) fractions were analyzed by SDS-PAGE followed by immunoblotting with anti-GFP antibody. (C) Quantitative comparison of the immunoprecipitation efficiency of wild-type and mutated (G161T, A188S and T189A) hPARP1 ZnF2 with the PARP1 nanotrap. Quantitative analysis of the signal densities on western blot was performed with ImageJ; the signals in the bound lanes were normalized to the input. Chart bars show mean ± S.D., T-test; * p<0.05; n = 4.
(JPG)

S2 Fig On-bead pADPr chain synthesis with immunoprecipitated endogenous hPARP1 after pull-down with the PARP1 nanotrap. Absorbance spectra of synthesized and purified pADPr polymers are shown. Reaction with the purified recombinant hPARP1 with NAD+ (A) or without NAD+ (B); on-bead reaction with PARP1 nanotrap-precipitated endogenous hPARP1 with NAD+ (C) or without NAD+ (D); on-bead reaction with unrelated nanotrap (GFP-Trap) with NAD+ (E) or without NAD+ (F) after IP.
(JPG)

S3 Fig PARP1 chromobody expression in living cells does not significantly affect cell viability or PARP1 enzymatic activity. (A) Comparison of the metabolic viability of HeLa cells transiently expressing PARP1 chromobody and untransfected HeLa cells (nt) in resazurin assay (alamarBlue). Percentages of viable cells after 24 h of proliferation were determined. Chart bars show mean ± S.D., no significant differences, n = 3. (B) pADPr immunostaining (in green) of HeLa cells stably expressing the PARP1 chromobody (in red) and HeLa cells without chromobody (C) after treatment with 10 mM H_2_O_2_ for 10 minutes. Scale bar, 10 μm.
(JPG)

S4 Fig High content analysis of compound potencies to delocalize hPARP1 from nucleoli as determined based on the PARP1 chromobody signal. HeLa cells stably expressing PARP1 chromobody were treated with different concentrations of 4-NQO, actinomycin D, camptothecin and H_2_O_2_ (titration series from 0.01 μM up to 1 mM) for 4 h. Subsequently, cells were fixed with 4% formaldehyde, counterstained for DAPI, imaged and analyzed in an automated fashion with the IN Cell Analyzer software. Segmentation analysis was applied and cells with/without PARP1 in nucleoli were counted in each well. Chart bars are mean ± S.D., T-test; *** p<0.005; n = 6.
(JPG)

S5 Fig Monitoring of laser-induced DNA damage with PARP1 chromobody in human and non-human cells. (A) Live-cell imaging of laser-microirradiated (405 nm laser, 100% power, 1 s) cells transiently expressing the PARP1 chromobody. Time-lapse imaging was carried out at 1 frame per second rate with a spinning disc microscope acquiring 9 pre-irradiation and 100 post-irradiation frames. Selected time-frames are shown, yellow circles depict the microirradiation regions (Ø 1 μM), yellow arrow-heads mark the sites before and after irradiation. Scale bar, 5 μm. (B) Quantitative evaluation of recruitment kinetics of endogenous hPARP1. Pre-irradiation intensity values were normalized to 100%, no correction for photobleaching during image acquisition was implemented. For each cell line, 10–14 cells were analyzed. Data are mean ± S.D.
(JPG)

S6 Fig Recruitment of GFP-hPARP1 and PARP1 chromobody to the DNA damage sites induced by laser microirradiation. HeLa cells transiently co-transfected with GFP-hPARP1 and PARP1 chromobody (TagRFP) were subjected to confocal imaging upon microirradiation with a 405 nm diode laser for 1 second. Time-lapse imaging was carried out at 1 frame per second rate with a spinning disc microscope. Scale bar, 5 μm.
(JPG)

S1 Video Live-cell movie of HeLa cells expressing PARP1 chromobody treated with 10 μM camptothecin followed by recovery. (GIF)

S2 Video Live-cell movie of laser-irradiated HeLa cells transiently expressing PARP1 chromobody. (GIF)

S3 Video Live-cell movie of carbon ion-irradiated HeLa cells transiently expressing PARP1 chromobody. (GIF)

## References

[1] Langelier MF, Servent KM, Rogers EE, Pascal JM. A third zinc-binding domain of human poly(ADP-ribose) polymerase-1 coordinates DNA-dependent enzyme activation. The Journal of biological chemistry. 2008;283(7):4105–14. Epub 2007/12/07. 10.1074/jbc.M708558200 . 18055453

[2] Tao Z, Gao P, Hoffman DW, Liu HW. Domain C of human poly(ADP-ribose) polymerase-1 is important for enzyme activity and contains a novel zinc-ribbon motif. Biochemistry. 2008;47(21):5804–13. Epub 2008/05/03. 10.1021/bi800018a . 18452307

[3] Kameshita I, Matsuda Z, Taniguchi T, Shizuta Y. Poly (ADP-Ribose) synthetase. Separation and identification of three proteolytic fragments as the substrate-binding domain, the DNA-binding domain, and the automodification domain. The Journal of biological chemistry. 1984;259(8):4770–6. Epub 1984/04/25. . 6325408

[4] de Murcia G, Menissier de Murcia J. Poly(ADP-ribose) polymerase: a molecular nick-sensor. Trends in biochemical sciences. 1994;19(4):172–6. Epub 1994/04/01. . 8016868 10.1016/0968-0004(94)90280-1

[5] Haince JF, McDonald D, Rodrigue A, Dery U, Masson JY, Hendzel MJ, et al PARP1-dependent kinetics of recruitment of MRE11 and NBS1 proteins to multiple DNA damage sites. The Journal of biological chemistry. 2008;283(2):1197–208. Epub 2007/11/21. 10.1074/jbc.M706734200 . 18025084

[6] Langelier MF, Pascal JM. PARP-1 mechanism for coupling DNA damage detection to poly(ADP-ribose) synthesis. Current opinion in structural biology. 2013;23(1):134–43. Epub 2013/01/22. 10.1016/j.sbi.2013.01.003 23333033 PMC3572337

[7] Schreiber V, Dantzer F, Ame JC, de Murcia G. Poly(ADP-ribose): novel functions for an old molecule. Nature reviews Molecular cell biology. 2006;7(7):517–28. Epub 2006/07/11. 10.1038/nrm1963 . 16829982

[8] Ogata N, Ueda K, Kawaichi M, Hayaishi O. Poly(ADP-ribose) synthetase, a main acceptor of poly(ADP-ribose) in isolated nuclei. The Journal of biological chemistry. 1981;256(9):4135–7. Epub 1981/05/10. . 6260786

[9] Tallis M, Morra R, Barkauskaite E, Ahel I. Poly(ADP-ribosyl)ation in regulation of chromatin structure and the DNA damage response. Chromosoma. 2014;123(1–2):79–90. 10.1007/s00412-013-0442-9 . 24162931

[10] Ekblad T, Camaioni E, Schuler H, Macchiarulo A. PARP inhibitors: polypharmacology versus selective inhibition. The FEBS journal. 2013;280(15):3563–75. Epub 2013/04/23. 10.1111/febs.12298 . 23601167

[11] Schnell U, Dijk F, Sjollema KA, Giepmans BN. Immunolabeling artifacts and the need for live-cell imaging. Nature methods. 2012;9(2):152–8. Epub 2012/02/01. 10.1038/nmeth.1855 . 22290187

[12] Stadler C, Rexhepaj E, Singan VR, Murphy RF, Pepperkok R, Uhlen M, et al Immunofluorescence and fluorescent-protein tagging show high correlation for protein localization in mammalian cells. Nature methods. 2013;10(4):315–23. Epub 2013/02/26. 10.1038/nmeth.2377 . 23435261

[13] Rancourt A, Satoh MS. Delocalization of nucleolar poly(ADP-ribose) polymerase-1 to the nucleoplasm and its novel link to cellular sensitivity to DNA damage. DNA repair. 2009;8(3):286–97. Epub 2009/01/16. 10.1016/j.dnarep.2008.11.018 . 19144573

[14] Meder VS, Boeglin M, de Murcia G, Schreiber V. PARP-1 and PARP-2 interact with nucleophosmin/B23 and accumulate in transcriptionally active nucleoli. Journal of cell science. 2005;118(Pt 1):211–22. Epub 2004/12/24. 10.1242/jcs.01606 . 15615785

[15] Desnoyers S, Kaufmann SH, Poirier GG. Alteration of the nucleolar localization of poly(ADP-ribose) polymerase upon treatment with transcription inhibitors. Experimental cell research. 1996;227(1):146–53. Epub 1996/08/25. 10.1006/excr.1996.0259 . 8806461

[16] Lamarre D, Talbot B, de Murcia G, Laplante C, Leduc Y, Mazen A, et al Structural and functional analysis of poly(ADP ribose) polymerase: an immunological study. Biochimica et biophysica acta. 1988;950(2):147–60. Epub 1988/07/13. . 2454668 10.1016/0167-4781(88)90007-3

[17] Traenkle B, Emele F, Anton R, Poetz O, Haeussler RS, Maier J, et al Monitoring interactions and dynamics of endogenous beta-catenin with intracellular nanobodies in living cells. Molecular & cellular proteomics: MCP. 2015;14(3):707–23. 10.1074/mcp.M114.044016 25595278 PMC4349989

[18] Rocchetti A, Hawes C, Kriechbaumer V. Fluorescent labelling of the actin cytoskeleton in plants using a cameloid antibody. Plant methods. 2014;10:12 10.1186/1746-4811-10-12 24872838 PMC4036722

[19] Zolghadr K, Gregor J, Leonhardt H, Rothbauer U. Case study on live cell apoptosis-assay using lamin-chromobody cell-lines for high-content analysis. Methods Mol Biol. 2012;911:569–75. 10.1007/978-1-61779-968-6_36 . 22886277

[20] Burgess A, Lorca T, Castro A. Quantitative live imaging of endogenous DNA replication in mammalian cells. PloS one. 2012;7(9):e45726 10.1371/journal.pone.0045726 23029203 PMC3447815

[21] Rothbauer U, Zolghadr K, Tillib S, Nowak D, Schermelleh L, Gahl A, et al Targeting and tracing antigens in live cells with fluorescent nanobodies. Nature methods. 2006;3(11):887–9. Epub 2006/10/25. 10.1038/nmeth953 . 17060912

[22] Arbabi Ghahroudi M, Desmyter A, Wyns L, Hamers R, Muyldermans S. Selection and identification of single domain antibody fragments from camel heavy-chain antibodies. FEBS letters. 1997;414(3):521–6. Epub 1997/10/10. . 9323027 10.1016/s0014-5793(97)01062-4

[23] Buchfellner A. Selection and characterization of an alpaca derived single domain antibody for biochemical and live cell analysis of human poly(ADP-ribose) polymerase 1 [Dissertation (Ph.D.)]: The Natural and Medical Sciences Institute at the University of Tübingen; 2014.

[24] Rothbauer U, Zolghadr K, Muyldermans S, Schepers A, Cardoso MC, Leonhardt H. A versatile nanotrap for biochemical and functional studies with fluorescent fusion proteins. Molecular & cellular proteomics: MCP. 2008;7(2):282–9. Epub 2007/10/24. 10.1074/mcp.M700342-MCP200 . 17951627

[25] Kiehlbauch CC, Aboul-Ela N, Jacobson EL, Ringer DP, Jacobson MK. High resolution fractionation and characterization of ADP-ribose polymers. Analytical biochemistry. 1993;208(1):26–34. Epub 1993/01/01. . 8434792 10.1006/abio.1993.1004

[26] Fahrer J. Interaction of Poly(ADP-ribose) and specific binding proteins as a function of chain length [Dissertation (Ph.D.)]. University of Konstanz 2007. 10.1093/nar/gkm944 PMC2175335 17991682

[27] Drexler GA, Siebenwirth C, Drexler SE, Girst S, Greubel C, Dollinger G, et al Live cell imaging at the Munich ion microbeam SNAKE—a status report. Radiat Oncol. 2015;10:42 10.1186/s13014-015-0350-7 25880907 PMC4341815

[28] Hable V. The live cell irradiation and observation setup at SNAKE. Nuclear Instruments and Methods in Physics Research Section B: Beam Interactions with Materials and Atoms. 2009;267(12–13):2090–7. 10.1016/j.nimb.2009.03.071

[29] Hauptner A, Dietzel S, Drexler GA, Reichart P, Krucken R, Cremer T, et al Microirradiation of cells with energetic heavy ions. Radiation and environmental biophysics. 2004;42(4):237–45. Epub 2004/01/22. 10.1007/s00411-003-0222-7 . 14735370

[30] Siebenwirth C. Determination of the accuracy for targeted irradiations of cellular substructures at SNAKE. Nuclear Instruments and Methods in Physics Research Section B: Beam Interactions with Materials and Atoms. 2015;348:137–42. 10.1016/j.nimb.2015.01.064

[31] Saxena A, Wong LH, Kalitsis P, Earle E, Shaffer LG, Choo KH. Poly(ADP-ribose) polymerase 2 localizes to mammalian active centromeres and interacts with PARP-1, Cenpa, Cenpb and Bub3, but not Cenpc. Human molecular genetics. 2002;11(19):2319–29. Epub 2002/09/10. . 12217960 10.1093/hmg/11.19.2319

[32] Schreiber V, Ame JC, Dolle P, Schultz I, Rinaldi B, Fraulob V, et al Poly(ADP-ribose) polymerase-2 (PARP-2) is required for efficient base excision DNA repair in association with PARP-1 and XRCC1. The Journal of biological chemistry. 2002;277(25):23028–36. Epub 2002/04/12. . 11948190 10.1074/jbc.M202390200

[33] Augustin A, Spenlehauer C, Dumond H, Menissier-De Murcia J, Piel M, Schmit AC, et al PARP-3 localizes preferentially to the daughter centriole and interferes with the G1/S cell cycle progression. Journal of cell science. 2003;116(Pt 8):1551–62. Epub 2003/03/18. . 12640039 10.1242/jcs.00341

[34] Rouleau M, McDonald D, Gagne P, Ouellet ME, Droit A, Hunter JM, et al PARP-3 associates with polycomb group bodies and with components of the DNA damage repair machinery. Journal of cellular biochemistry. 2007;100(2):385–401. Epub 2006/08/23. 10.1002/jcb.21051 . 16924674

[35] Zolghadr K, Mortusewicz O, Rothbauer U, Kleinhans R, Goehler H, Wanker EE, et al A fluorescent two-hybrid assay for direct visualization of protein interactions in living cells. Molecular & cellular proteomics: MCP. 2008;7(11):2279–87. Epub 2008/07/16. 10.1074/mcp.M700548-MCP200 . 18622019

[36] Yurlova L, Derks M, Buchfellner A, Hickson I, Janssen M, Morrison D, et al The fluorescent two-hybrid assay to screen for protein-protein interaction inhibitors in live cells: targeting the interaction of p53 with Mdm2 and Mdm4. Journal of biomolecular screening. 2014;19(4):516–25. Epub 2014/01/31. 10.1177/1087057113518067 . 24476585

[37] Yamanaka H, Willis EH, Penning CA, Peebles CL, Tan EM, Carson DA. Human autoantibodies to poly(adenosine diphosphate-ribose) polymerase. The Journal of clinical investigation. 1987;80(3):900–4. Epub 1987/09/01. 10.1172/JCI113150 2442198 PMC442319

[38] Thorslund T, von Kobbe C, Harrigan JA, Indig FE, Christiansen M, Stevnsner T, et al Cooperation of the Cockayne syndrome group B protein and poly(ADP-ribose) polymerase 1 in the response to oxidative stress. Molecular and cellular biology. 2005;25(17):7625–36. 10.1128/MCB.25.17.7625-7636.2005 16107709 PMC1190307

[39] Dantzer F, Nasheuer HP, Vonesch JL, de Murcia G, Menissier-de Murcia J. Functional association of poly(ADP-ribose) polymerase with DNA polymerase alpha-primase complex: a link between DNA strand break detection and DNA replication. Nucleic acids research. 1998;26(8):1891–8. 9518481 10.1093/nar/26.8.1891 PMC147507

[40] Yung TM, Sato S, Satoh MS. Poly(ADP-ribosyl)ation as a DNA damage-induced post-translational modification regulating poly(ADP-ribose) polymerase-1-topoisomerase I interaction. The Journal of biological chemistry. 2004;279(38):39686–96. Epub 2004/07/13. 10.1074/jbc.M402729200 . 15247263

[41] Wang HK, Morris-Natschke SL, Lee KH. Recent advances in the discovery and development of topoisomerase inhibitors as antitumor agents. Medicinal research reviews. 1997;17(4):367–425. Epub 1997/07/01. . 9211397 10.1002/(sici)1098-1128(199707)17:4<367::aid-med3>3.0.co;2-u

[42] Redinbo MR, Stewart L, Kuhn P, Champoux JJ, Hol WG. Crystal structures of human topoisomerase I in covalent and noncovalent complexes with DNA. Science. 1998;279(5356):1504–13. Epub 1998/03/21. . 9488644 10.1126/science.279.5356.1504

[43] Perry RP, Kelley DE. Inhibition of RNA synthesis by actinomycin D: characteristic dose-response of different RNA species. Journal of cellular physiology. 1970;76(2):127–39. Epub 1970/10/01. 10.1002/jcp.1040760202 . 5500970

[44] Shanmugam G, Bhargava PM. The effect of actinomycin D on the synthesis of ribonucleic acid and protein in rat liver parenchymal cells in suspension and liver slices. The Biochemical journal. 1968;108(5):741–8. Epub 1968/08/01. 5673524 10.1042/bj1080741 PMC1198879

[45] Watson WE. Observations on the nucleolar and total cell body nucleic acid of injured nerve cells. The Journal of physiology. 1968;196(3):655–76. Epub 1968/06/01. 5664236 10.1113/jphysiol.1968.sp008528 PMC1351769

[46] Sobell HM. Actinomycin and DNA transcription. Proceedings of the National Academy of Sciences of the United States of America. 1985;82(16):5328–31. Epub 1985/08/01. 2410919 10.1073/pnas.82.16.5328 PMC390561

[47] Yu YN, Ding C, Cai ZN, Chen XR. Cell cycle effects on the basal and DNA-damaging-agent-stimulated ADPRT activity in cultured mammalian cells. Mutation research. 1986;174(3):233–9. Epub 1986/07/01. . 3088445 10.1016/0165-7992(86)90157-0

[48] Gray MD, Wang L, Youssoufian H, Martin GM, Oshima J. Werner helicase is localized to transcriptionally active nucleoli of cycling cells. Experimental cell research. 1998;242(2):487–94. Epub 1998/07/31. 10.1006/excr.1998.4124 . 9683536

[49] Wu RS, Kumar A, Warner JR. Ribosome formation is blocked by camptothecin, a reversible inhibitor of RNA synthesis. Proceedings of the National Academy of Sciences of the United States of America. 1971;68(12):3009–14. 5289246 10.1073/pnas.68.12.3009 PMC389580

[50] Yung BY, Bor AM, Chan PK. Short exposure to actinomycin D induces "reversible" translocation of protein B23 as well as "reversible" inhibition of cell growth and RNA synthesis in HeLa cells. Cancer research. 1990;50(18):5987–91. Epub 1990/09/15. . 1697505

[51] Snyderwine EG, Bohr VA. Gene- and strand-specific damage and repair in Chinese hamster ovary cells treated with 4-nitroquinoline 1-oxide. Cancer research. 1992;52(15):4183–9. Epub 1992/08/01. . 1638532

[52] Kielbassa C, Roza L, Epe B. Wavelength dependence of oxidative DNA damage induced by UV and visible light. Carcinogenesis. 1997;18(4):811–6. Epub 1997/04/01. . 9111219 10.1093/carcin/18.4.811

[53] Ferrando-May E, Tomas M, Blumhardt P, Stockl M, Fuchs M, Leitenstorfer A. Highlighting the DNA damage response with ultrashort laser pulses in the near infrared and kinetic modeling. Frontiers in genetics. 2013;4:135 Epub 2013/07/25. 10.3389/fgene.2013.00135 23882280 PMC3712194

[54] Splinter J, Jakob B, Lang M, Yano K, Engelhardt J, Hell SW, et al Biological dose estimation of UVA laser microirradiation utilizing charged particle-induced protein foci. Mutagenesis. 2010;25(3):289–97. 10.1093/mutage/geq005 20167590 PMC2902920

[55] Vignard J, Mirey G, Salles B. Ionizing-radiation induced DNA double-strand breaks: a direct and indirect lighting up. Radiotherapy and oncology: journal of the European Society for Therapeutic Radiology and Oncology. 2013;108(3):362–9. 10.1016/j.radonc.2013.06.013 . 23849169

[56] Seiler DM, Rouquette J, Schmid VJ, Strickfaden H, Ottmann C, Drexler GA, et al Double-strand break-induced transcriptional silencing is associated with loss of tri-methylation at H3K4. Chromosome research: an international journal on the molecular, supramolecular and evolutionary aspects of chromosome biology. 2011;19(7):883–99. Epub 2011/10/12. 10.1007/s10577-011-9244-1 . 21987186

[57] De Genst E, Silence K, Decanniere K, Conrath K, Loris R, Kinne J, et al Molecular basis for the preferential cleft recognition by dromedary heavy-chain antibodies. Proceedings of the National Academy of Sciences of the United States of America. 2006;103(12):4586–91. Epub 2006/03/16. 10.1073/pnas.0505379103 16537393 PMC1450215

[58] Nunes-Silva S, Gangnard S, Vidal M, Vuchelen A, Dechavanne S, Chan S, et al Llama immunization with full-length VAR2CSA generates cross-reactive and inhibitory single-domain antibodies against the DBL1X domain. Scientific reports. 2014;4:7373 10.1038/srep07373 . 25487735 PMC5376981

[59] Bethuyne J, De Gieter S, Zwaenepoel O, Garcia-Pino A, Durinck K, Verhelle A, et al A nanobody modulates the p53 transcriptional program without perturbing its functional architecture. Nucleic acids research. 2014 Epub 2014/10/18. 10.1093/nar/gku962 . 25324313 PMC4227789

[60] Jobling SA, Jarman C, Teh MM, Holmberg N, Blake C, Verhoeyen ME. Immunomodulation of enzyme function in plants by single-domain antibody fragments. Nature biotechnology. 2003;21(1):77–80. Epub 2002/12/17. 10.1038/nbt772 . 12483224

[61] Oyen D, Srinivasan V, Steyaert J, Barlow JN. Constraining enzyme conformational change by an antibody leads to hyperbolic inhibition. Journal of molecular biology. 2011;407(1):138–48. Epub 2011/01/18. 10.1016/j.jmb.2011.01.017 . 21238460

[62] Hermans P, Adams H, Detmers F. Purification of antibodies and antibody fragments using CaptureSelect affinity resins. Methods Mol Biol. 2014;1131:297–314. 10.1007/978-1-62703-992-5_19 . 24515474

[63] Leonhardt H, Rahn HP, Cardoso MC. Intranuclear targeting of DNA replication factors. Journal of cellular biochemistry Supplement. 1998;30–31:243–9. Epub 1999/01/20. . 9893277

[64] Fakan S, Leduc Y, Lamarre D, Brunet G, Poirier GG. Immunoelectron microscopical distribution of poly(ADP-ribose)polymerase in the mammalian cell nucleus. Experimental cell research. 1988;179(2):517–26. Epub 1988/12/01. . 3142785 10.1016/0014-4827(88)90289-3

[65] de Murcia G, Huletsky A, Poirier GG. Modulation of chromatin structure by poly(ADP-ribosyl)ation. Biochemistry and cell biology = Biochimie et biologie cellulaire. 1988;66(6):626–35. Epub 1988/06/01. . 3139015 10.1139/o88-072

[66] Kaufmann SH, Brunet G, Talbot B, Lamarr D, Dumas C, Shaper JH, et al Association of poly(ADP-ribose) polymerase with the nuclear matrix: the role of intermolecular disulfide bond formation, RNA retention, and cell type. Experimental cell research. 1991;192(2):524–35. Epub 1991/02/01. . 1703086 10.1016/0014-4827(91)90072-3

[67] Mortusewicz O, Ame JC, Schreiber V, Leonhardt H. Feedback-regulated poly(ADP-ribosyl)ation by PARP-1 is required for rapid response to DNA damage in living cells. Nucleic acids research. 2007;35(22):7665–75. Epub 2007/11/06. 10.1093/nar/gkm933 17982172 PMC2190722

