# Supplementary figures and images for: A New Nanobody-Based Biosensor to Study Endogenous PARP1 In Vitro and in Live Human Cells
Source: PLoS One. 2016 Mar 7;11(3):e0151041. doi: 10.1371/journal.pone.0151041 (PMC4780744; doi:10.1371/journal.pone.0151041)

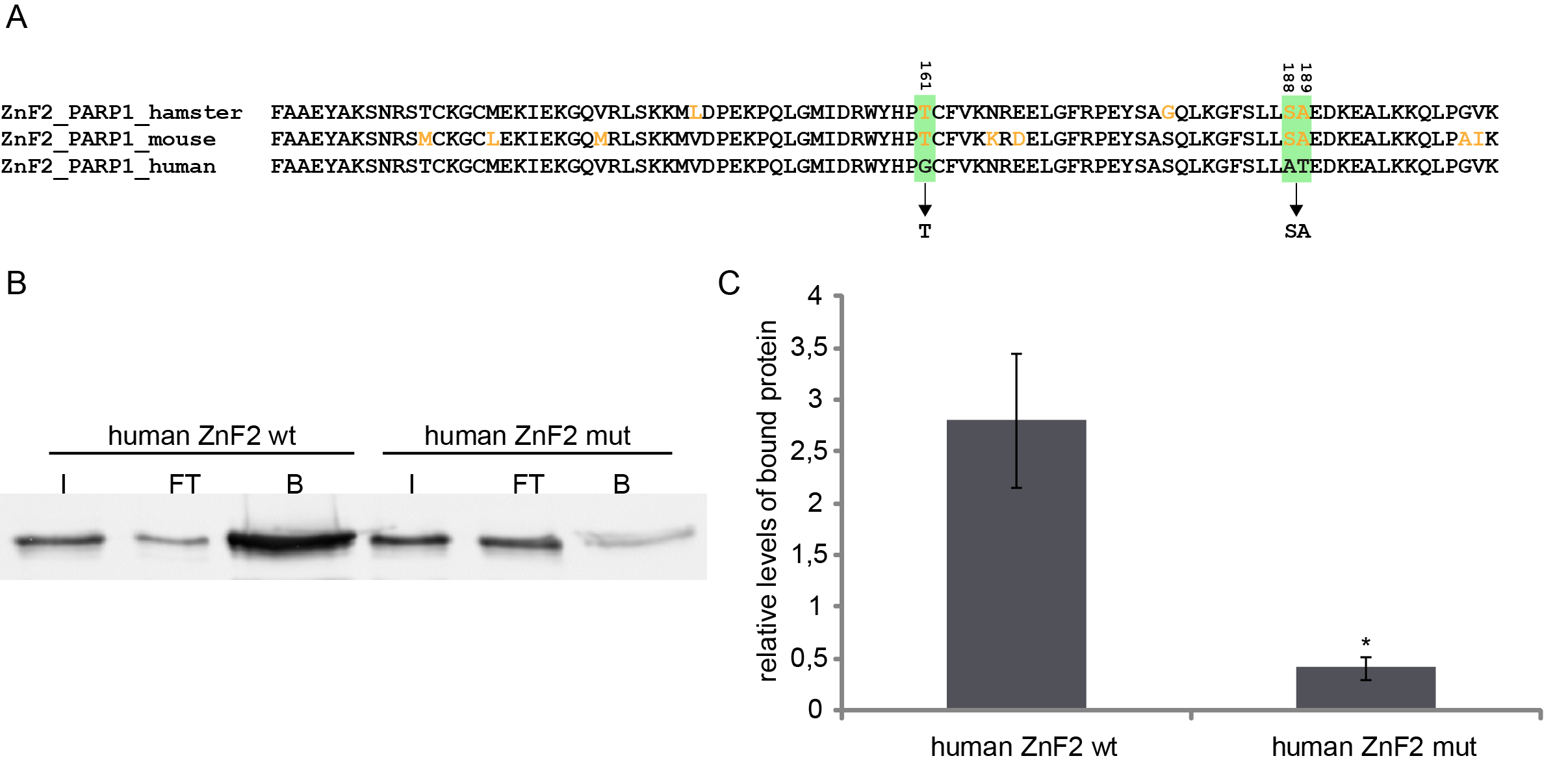

Supplement: S1 Fig — (A) Amino-acid sequence alignment of the PARP1 ZnF2 domains from hamster (Mesocricetus auratus, NCBI Reference Sequence: XP_005078195.1), mouse (Mus musculus, NCBI Reference Sequence: NP_031441.2) and human (Homo sapiens, NCBI Reference Sequence: NP_001609.2). Differences with respect to the human amino acid sequence are highlighted in yellow. Positions for the introduced single mutations are highlighted in green. (B) HEK293T cells were transiently transfected with the GFP-tagged wild-type or mutated (G161T, A188S and T189A) ZnF2 domain of hPARP1, lysed and subjected to pull-down with the PARP1 nanotrap. Input (I), flow-through (FT) and bound (B) fractions were analyzed by SDS-PAGE followed by immunoblotting with anti-GFP antibody. (C) Quantitative comparison of the immunoprecipitation efficiency of wild-type and mutated (G161T, A188S and T189A) hPARP1 ZnF2 with the PARP1 nanotrap. Quantitative analysis of the signal densities on western blot was performed with ImageJ; the signals in the bound lanes were normalized to the input. Chart bars show mean ± S.D., T-test; * p<0.05; n = 4. (JPG) [file pone.0151041.s001.jpg]

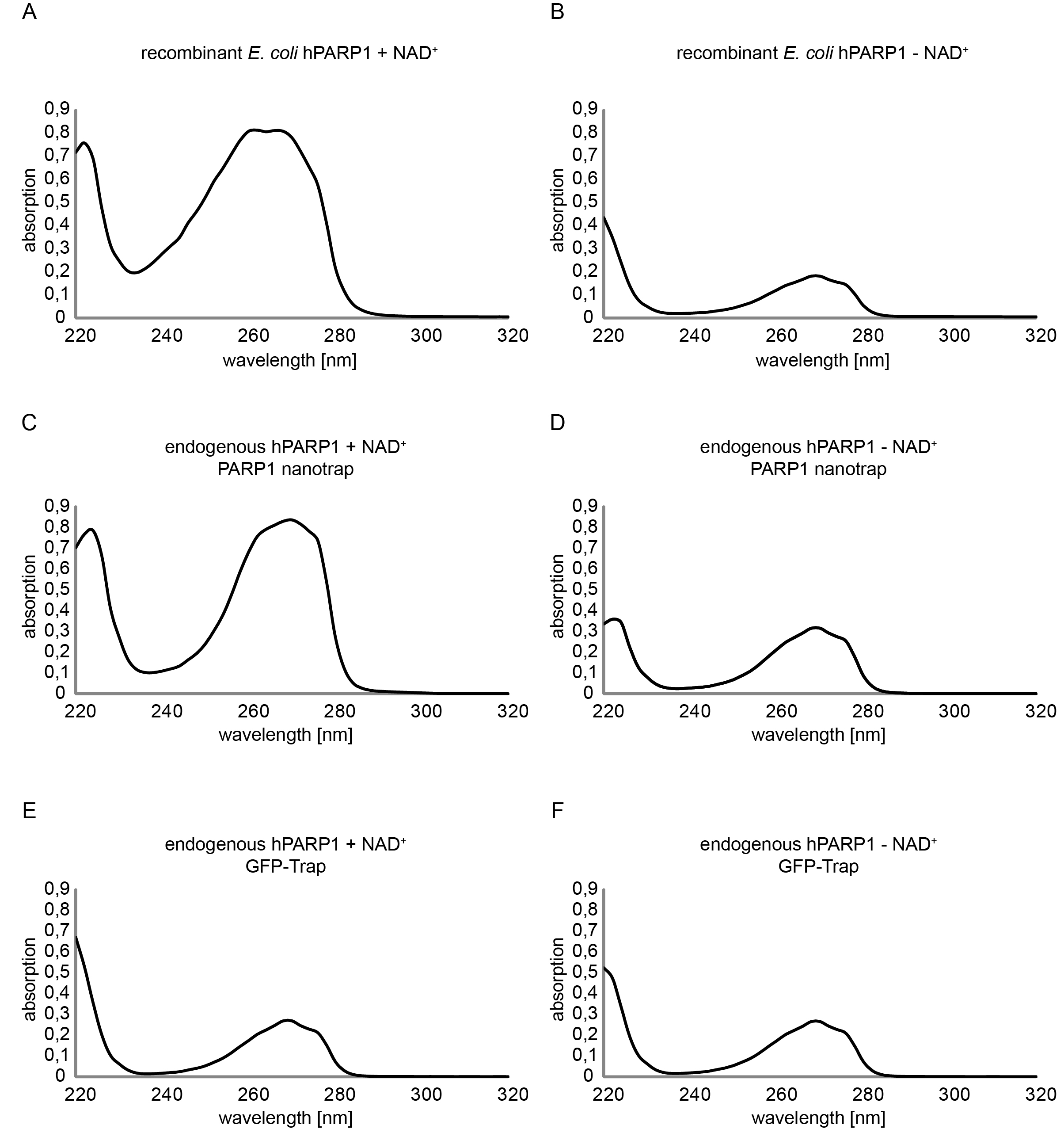

Supplement: S2 Fig — Absorbance spectra of synthesized and purified pADPr polymers are shown. Reaction with the purified recombinant hPARP1 with NAD+ (A) or without NAD+ (B); on-bead reaction with PARP1 nanotrap-precipitated endogenous hPARP1 with NAD+ (C) or without NAD+ (D); on-bead reaction with unrelated nanotrap (GFP-Trap) with NAD+ (E) or without NAD+ (F) after IP. (JPG) [file pone.0151041.s002.jpg]

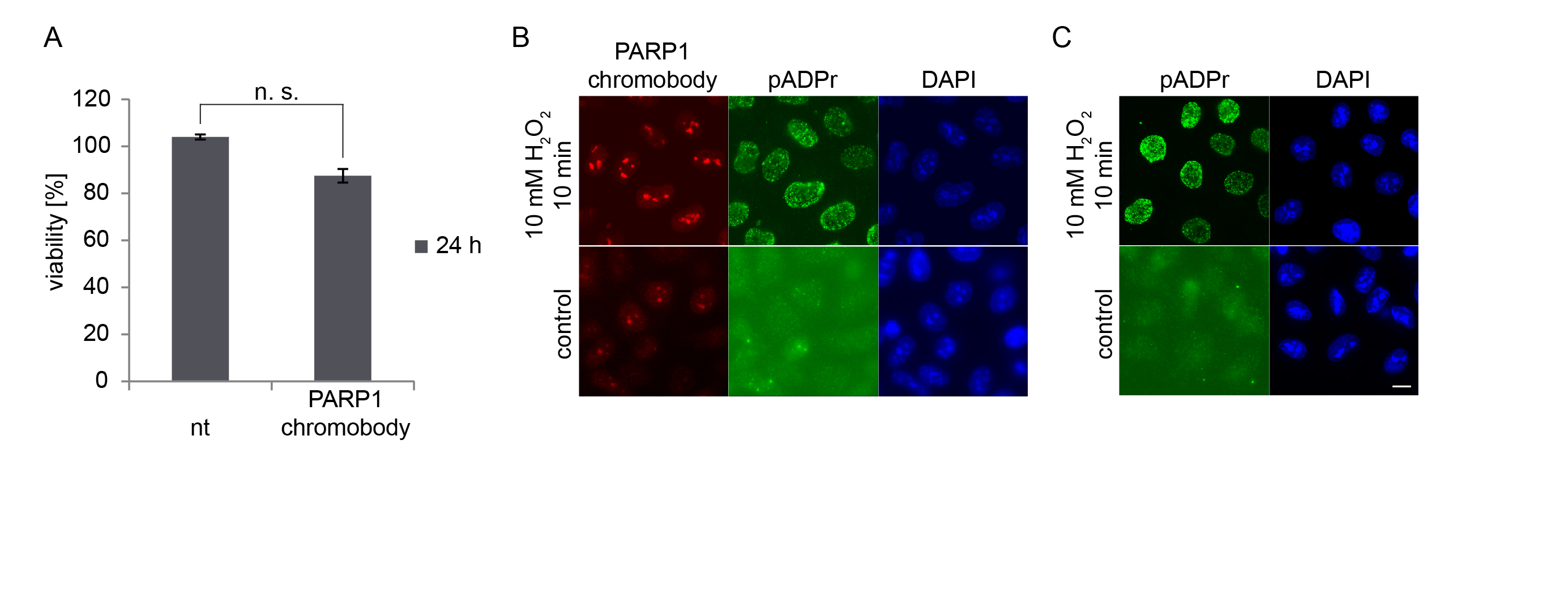

Supplement: S3 Fig — (A) Comparison of the metabolic viability of HeLa cells transiently expressing PARP1 chromobody and untransfected HeLa cells (nt) in resazurin assay (alamarBlue). Percentages of viable cells after 24 h of proliferation were determined. Chart bars show mean ± S.D., no significant differences, n = 3. (B) pADPr immunostaining (in green) of HeLa cells stably expressing the PARP1 chromobody (in red) and HeLa cells without chromobody (C) after treatment with 10 mM H2O2 for 10 minutes. Scale bar, 10 μm. (JPG) [file pone.0151041.s003.jpg]

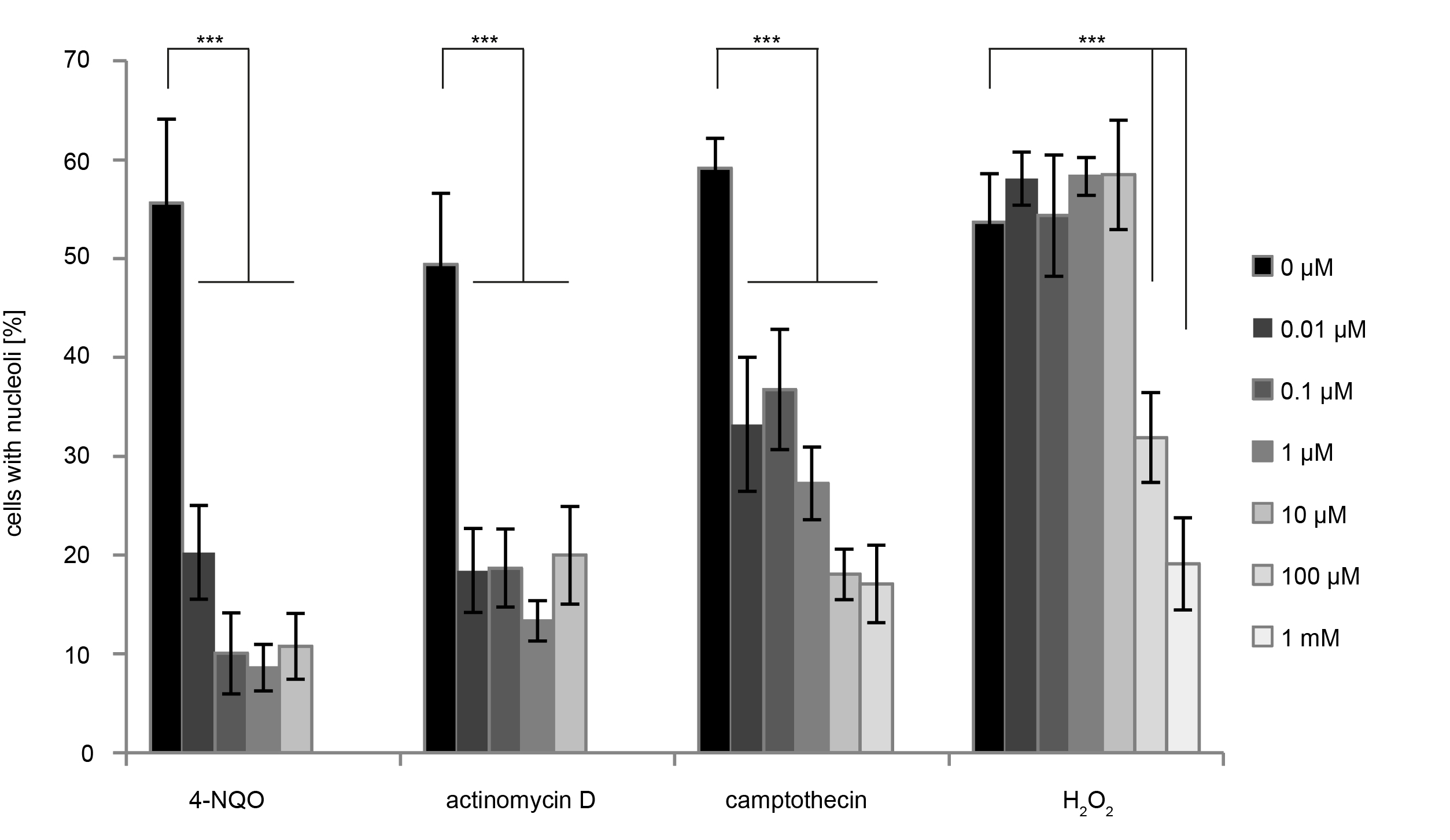

Supplement: S4 Fig — HeLa cells stably expressing PARP1 chromobody were treated with different concentrations of 4-NQO, actinomycin D, camptothecin and H2O2 (titration series from 0.01 μM up to 1 mM) for 4 h. Subsequently, cells were fixed with 4% formaldehyde, counterstained for DAPI, imaged and analyzed in an automated fashion with the IN Cell Analyzer software. Segmentation analysis was applied and cells with/without PARP1 in nucleoli were counted in each well. Chart bars are mean ± S.D., T-test; *** p<0.005; n = 6. (JPG) [file pone.0151041.s004.jpg]

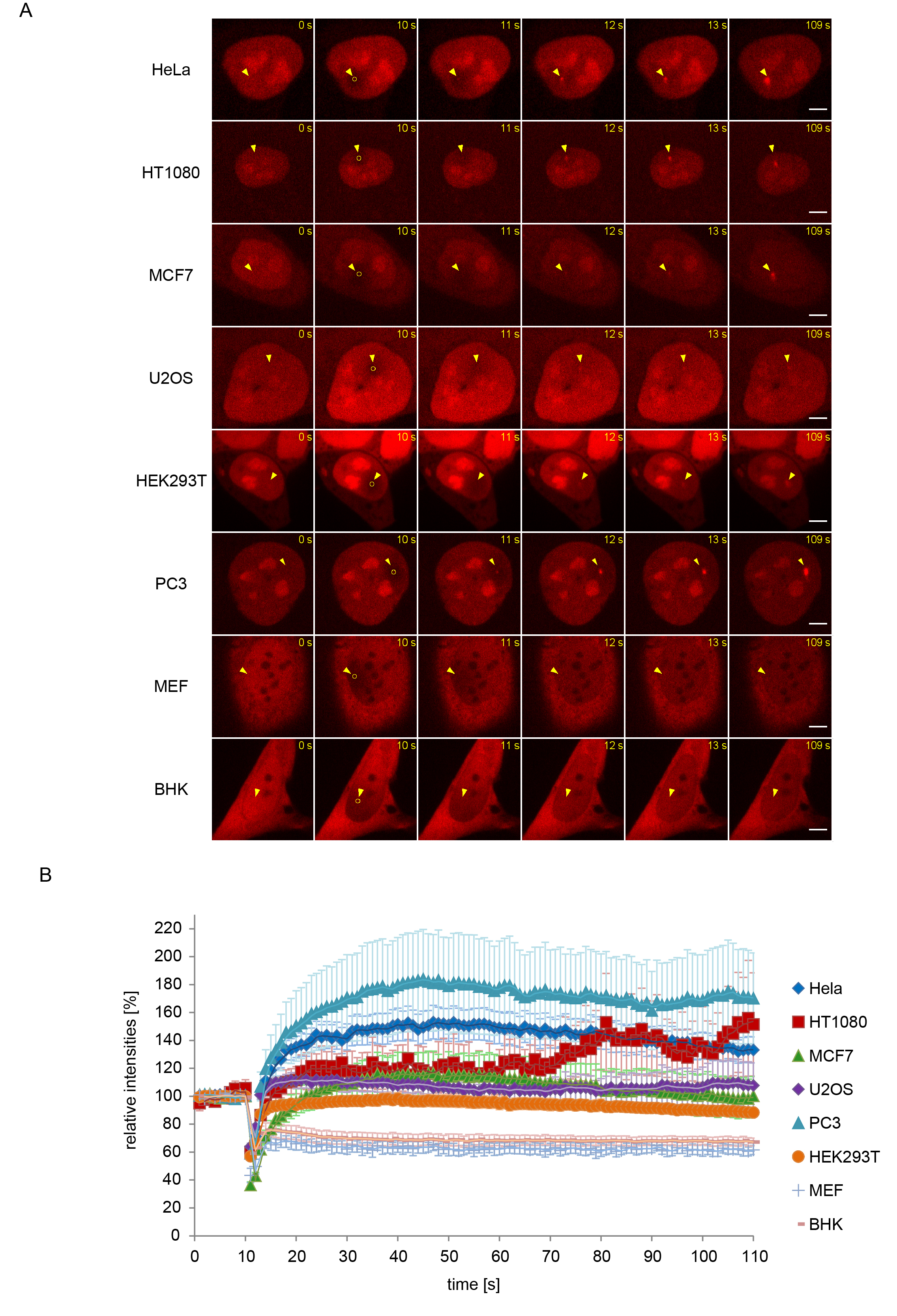

Supplement: S5 Fig — (A) Live-cell imaging of laser-microirradiated (405 nm laser, 100% power, 1 s) cells transiently expressing the PARP1 chromobody. Time-lapse imaging was carried out at 1 frame per second rate with a spinning disc microscope acquiring 9 pre-irradiation and 100 post-irradiation frames. Selected time-frames are shown, yellow circles depict the microirradiation regions (Ø 1 μM), yellow arrow-heads mark the sites before and after irradiation. Scale bar, 5 μm. (B) Quantitative evaluation of recruitment kinetics of endogenous hPARP1. Pre-irradiation intensity values were normalized to 100%, no correction for photobleaching during image acquisition was implemented. For each cell line, 10–14 cells were analyzed. Data are mean ± S.D. (JPG) [file pone.0151041.s005.jpg]

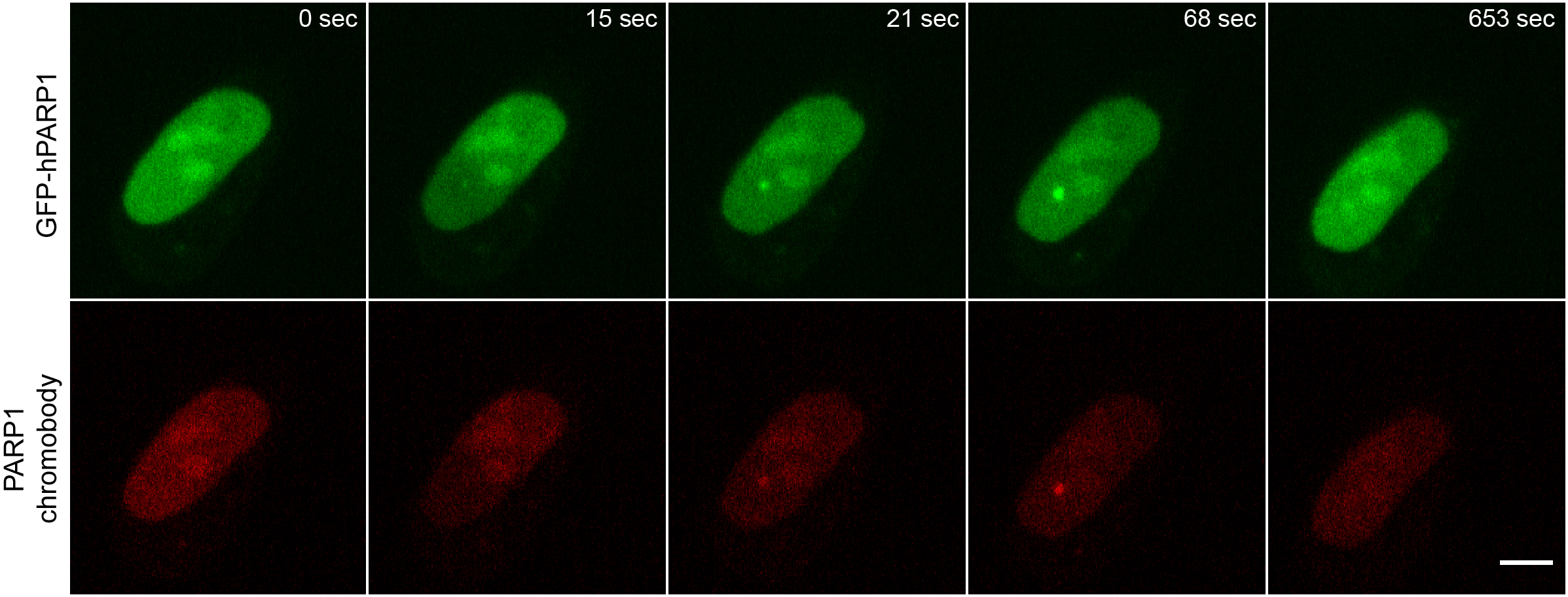

Supplement: S6 Fig — HeLa cells transiently co-transfected with GFP-hPARP1 and PARP1 chromobody (TagRFP) were subjected to confocal imaging upon microirradiation with a 405 nm diode laser for 1 second. Time-lapse imaging was carried out at 1 frame per second rate with a spinning disc microscope. Scale bar, 5 μm. (JPG) [file pone.0151041.s006.jpg]

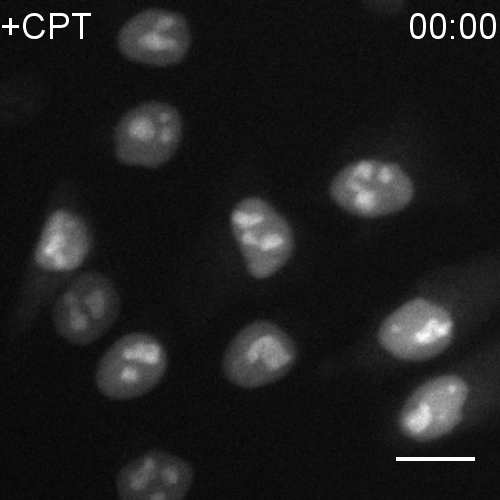

Supplement: S1 Video — (GIF) [file pone.0151041.s007.gif]

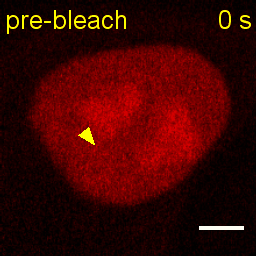

Supplement: S2 Video — (GIF) [file pone.0151041.s008.gif]

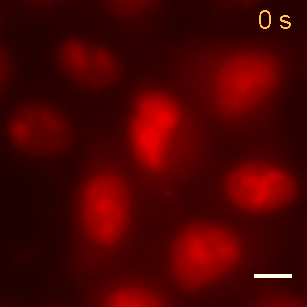

Supplement: S3 Video — (GIF) [file pone.0151041.s009.gif]
